# Atg7 senses ATP levels and regulates AKT_1_-PDCD4 phosphorylation-ubiquitination axis to promote survival during metabolic stress

**DOI:** 10.1038/s42003-023-05656-7

**Published:** 2023-12-11

**Authors:** Chengsi Deng, Chunlu li, Xiang Dong, Yang Yu, Wendong Guo, Yi Guan, Xun Sun, Liu Cao

**Affiliations:** 1 Health Sciences Institute, College of Basic Medical Sciences, China Medical University, Shenyang, China; 2 Key Laboratory of Medical Cell Biology, Key Laboratory of Precision Diagnosis and Treatment of Gastrointestinal Tumors, Ministry of Education, China Medical University, Shenyang, China; 3 Department of Immunology, China Medical University, No.77 Puhe Road, Shenyang North New Area, Shenyang, Liaoning Province China

**Keywords:** Apoptosis, Stress signalling

## Abstract

We report that autophagy-related gene 7 (ATG7) modulates p53 activity to regulate cell cycle and survival during metabolic stress, and that indicates Atg7 is functionally involved in cellular homeostasis in autophagy independent fashion. As a protein translation inhibitor, Programmed cell death 4 (PDCD4) expression is regulated by AKT_1_ phosphorylation. Here, we find that Atg7 interacts with PDCD4 and AKT_1_ to regulate AKT_1_-PDCD4 phosphorylation-ubiquitination axis during metabolic stress. We demonstrate that Atg7 senses decrease of ATP levels to suppress AKT-mediated PDCD4 phosphorylation at Ser67, which inhibits PDCD4 ubiquitinating during metabolic stress. Finally, PDCD4 accumulates and functions as a protein translation inhibitor to conserve energy, thus reducing apoptosis and allowing cells to survive stress periods. These results suggest that the ATP-Atg7-PDCD4 axis acts as a metabolic adaptation pathway which dictates cells to overcome metabolic stress.

## Abstract (web summary)

This study reveals that ATG7, beyond its established role in autophagy, interacts with PDCD4 and AKT1 to regulate translation during metabolic stress. The ATG7PDCD4 axis operates as a metabolic adaptation pathway, conserving energy and fostering cell survival under stress.

## Introduction

Programmed cell death protein 4 (PDCD4) was discovered and named by Shibahara et al. in their exploration of apoptosis^1^. In recent studies, PDCD4 functions as a protein translation inhibitor^2,3^. Through the MA3 domain in its structure, PDCD4 interacts with eIF4A to control the synthesis of proteins, such as E-cadherin^4^, procaspase-3^5^, p21^6^, and p53^7^. The translational targets of PDCD4 are involved in cell proliferation and survival. The high levels of PDCD4 inhibit the proliferation of several cancer cell lines, including colon^8^, glioma^9^, breast^10^, and Medullary Thyroid Cancer^11^. In recent years, an increasing number of studies have been conducted on PDCD4 to investigate metabolism-related diseases, such as the PDCD4 regulation of insulin resistance^12,13^ and lipid metabolism disorders^14,15^. The involvement of PDCD4 in metabolic regulation has also been suggested by several researchers.

Autophagy is a lysosomal degradation pathway that degrades dysfunctional proteins, lipids, and organelles to maintain cellular homeostasis^16^. A decrease in the intracellular ATP level initiates autophagy^17,18^, which acts as a recycling mechanism that generates ATP to meet the intracellular energy requirements for cell survival^19^. The ability of autophagy-related proteins to act as ATP sensors to initiate autophagy has not been confirmed to date. Hence, we focus on Atg7, which has an adenylated structural domain at the C-terminus that contains the ubiquitin-like protein binding and adenylation active sites. Mutations in the adenylated domain cause Atg7 to lose its ability to activate Atg8 and transmit Atg8 to Atg3^20,21^, which are crucial steps in autophagy initiation. Therefore, ATP sensing by the adenylated structural domain of Atg7 is an important topic for further research.

Atg7-dependent autophagy is immediately activated upon starvation. Atg7 participates in the crucial stages of autophagy to recycle and reuse metabolites. Further, Atg7 interacts with p53 to regulate p21 to arrest the cell cycle and cell proliferation for reducing energy consumption and eventually, saving energy^22^. Thus, Atg7 helps the cells to survive starvation. PDCD4 protein levels are also significantly increased during ROS^23,24^. However, reports on whether the increased PDCD4 is involved in cell survival under ROS or nutrients withdraw are still lacking. In the present study, we investigated whether Atg7 can sense ATP deficiency and interact with PDCD4 to reduce protein synthesis, thus limiting ATP depletion.

Herein, the interaction between Atg7 and PDCD4 and the effect of the Atg7-PDCD4 pathway on cell survival during starvation were focused on. First, Atg7 was found to promote PDCD4 phosphorylation via the AKT pathway, and then trigger PDCD4 ubiquitination and subsequent degradation. Under normal conditions, the adenylated domain on Atg7 senses ATP and promotes AKT phosphorylation of PDCD4. This renders PDCD4 more readily phosphorylated for proteasome degradation and maintains PDCD4 at lower levels. During starvation, Atg7 senses ATP scarcity and decreases the interaction with PDCD4 to reduce its degradation. The increased PDCD4 level acts as a biochemical function to inhibit protein translation and reduce energy consumption, thus allowing the cells to safely survive starvation.

## Results

### Atg7 interacts with PDCD4 and negatively regulates PDCD4 protein levels

**Fig. 1:**
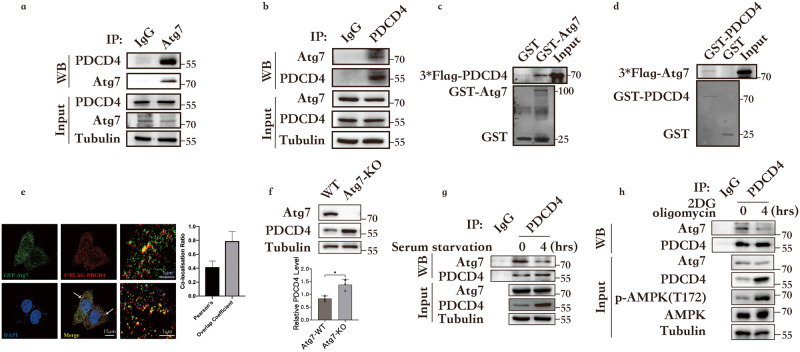
Atg7 interacts with PDCD4 and negatively regulates PDCD4 protein levels. **a**, **b** Co-immunoprecipitation assay was performed to detect the interaction between Atg7 and PDCD4. Lysates were extracted for immunoprecipitation with Atg7 (A) antibody or PDCD4 (B) antibody. **c** Glutathione S-transferase (GST) or GST-fused Atg7 protein and in vitro translated 3*Flag-PDCD4 were incubated for four hours, and the reaction was analyzed by SDS-PAGE. **d** Glutathione S-transferase (GST) or GST fused PDCD4 protein and in vitr*o* translated 3*Flag-Atg7 were incubated for four hours, and the reaction was analyzed by SDS-PAGE. **e** Co-transfected GFP-Atg7 and 3*Flag-PDCD4 plasmids in HEK293 cells, followed by immunofluorescence experiments. The Pearson’s correlation and overlap coefficient are shown in bar graph format (*n* = 20 cells) from three independent experiments were analyzed. **f** PDCD4 expression levels were increased in HCT116 Atg7-KO cells compared with that in Atg7-WT cells. Data represent the mean ± SD of three independent experiments (*n* = 3). *P* values were calculated by *t* test. *P < 0.05. **g** Interaction between endogenous Atg7 and PDCD4 in HCT116 cells under serum starvation for four hours. **h** Interaction between endogenous Atg7 and PDCD4 in HCT116 cells under ATP depletion induced by 2DG (5 mM) and oligomycin (2.5 μM) treatment for four hours.

Previous studies have demonstrated the role of Atg7 in maintaining metabolic homeostasis by initiating autophagy and arresting the cell cycle^17,18,22^. Herein, the function of Atg7 was more comprehensively investigated and it was determined whether Atg7 reduces protein synthesis to save ATP. The proteins interacting with Atg7 were analyzed by mass spectrometry, consequent to which the protein translation inhibitor PDCD4 was noticed. The co-immunoprecipitation assay was performed to detect the interaction between Atg7 and PDCD4. Endogenous Atg7 was found to interact with endogenous PDCD4 (Fig. 1a, b). The findings from the GST-pull-down assay confirmed that Atg7 directly interacted with PDCD4 (Fig. 1c, d). To clarify the sub-localizations of Atg7 and PDCD4 in HEK293 cells, GFP-Atg7 and 3*Flag-PDCD4 plasmids were co-transfected in HEK293 cells, followed by immunofluorescence experiments. The DeltaVision OMX 3D-SIM images show that Atg7 and PDCD4 were co-localized in the cellular matrix (Fig. 1e). This co-localization provides the spatial foundation for their binding.

In further experiments, the CRISPR-Cas9 system was used to construct Atg7 knockout cell lines to completely eliminate the interaction between PDCD4 and Atg7. The deletion of Atg7 was found to upregulate PDCD4 expression (Fig. 1f). To further verify the aforementioned phenomenon, Atg7 was overexpressed in the cells and this overexpression was found to downregulate PDCD4 expression (Supplementary Fig. 1c). Furthermore, qPCR results showed that *Atg7* knockdown or overexpression did not affect *PDCD4* expression at the mRNA level (Supplementary Fig. 1d, e). Therefore, we concluded that Atg7 downregulated PDCD4 expression at protein levels.

We further hypothesized that the reduction of Atg7-PDCD4 binding is associated with an increase in PDCD4 protein levels. The cells were subjected to starvation, which can induce autophagy. After four hours of starvation, PDCD4 was found to be increased to the maximum level (Supplementary Fig. 1a, b). Thereafter, the cells were cultured under starvation to determine any reduction in Atg7-PDCD4 binding. After serum starvation for four hours, the interaction between Atg7 and PDCD4 was found to be attenuated (Fig. 1g). Further, 2DG and oligomycin were applied to induce ATP depletion and this depletion was found to diminish the interaction between Atg7 and PDCD4 (Fig. 1h). These findings suggest that ATP acts as the signal that triggers PDCD4 upregulation and the attenuation of Atg7-PDCD4 interaction can be considered as the response to this signal.

### Atg7 degrades PDCD4 through the ubiquitin-proteasomal pathway

**Fig. 2:**
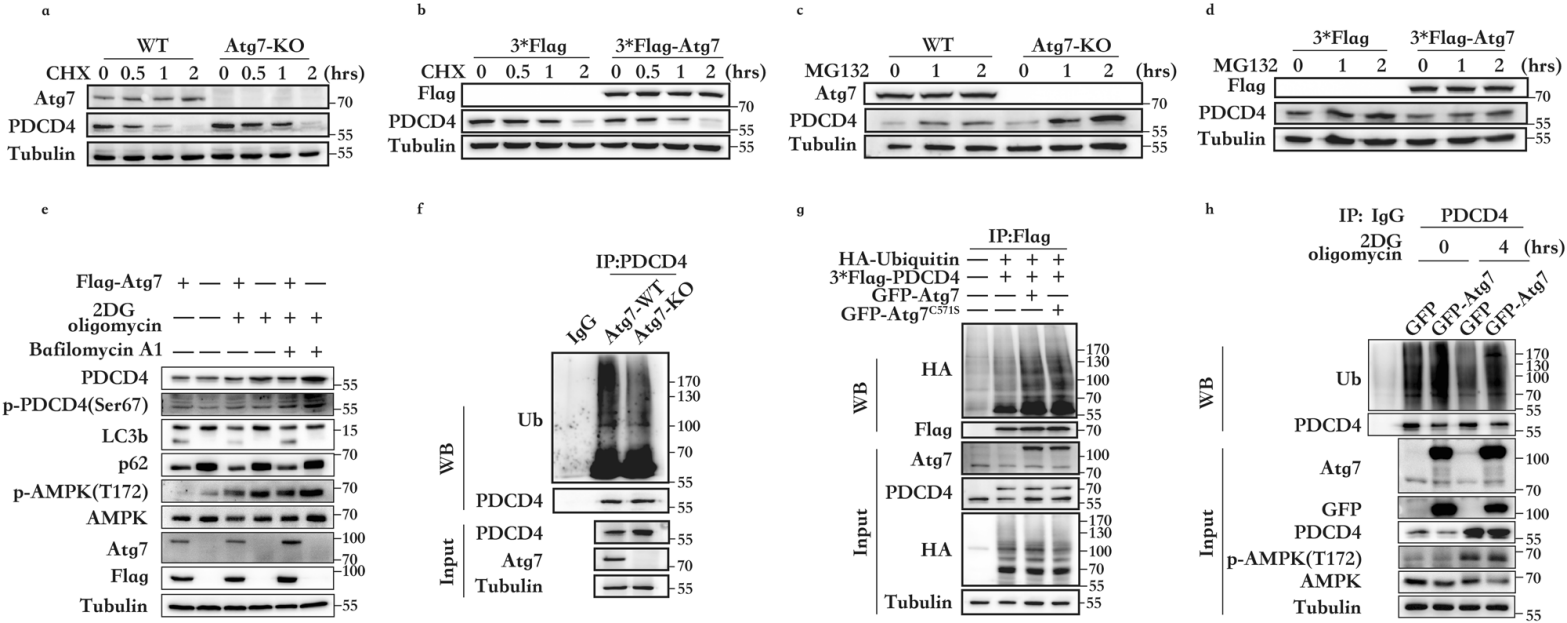
Atg7 degrades PDCD4 through the ubiquitin-proteasomal pathway. **a** PDCD44 half-life is extended in HEK293 Atg7-KO cells compared with that in Atg7-WT cells. The cells were switched to fresh medium (10% FBS) containing 50 mg/mL cycloheximide (CHX) for the indicated time and harvested for Western blot assay. **b** PDCD44 half-life is shortened in the HEK293 cells that were transfected with Flag-Atg7 compared to those transfected with the control vector. The cells were switched to fresh medium (10% FBS) containing 50 mg/mL cycloheximide (CHX) for the indicated time and harvested for Western blot assay. **c** Western blot detection of PDCD4 in HEK293 Atg7-KO cells andAtg7-WT cells after incubating with 10 mmol/L MG132 for the indicated times. **d** Western blot detection of PDCD4 in HEK293 cells that were transfected with Flag-Atg7 or control vector incubating with 10 mmol/L MG132 for the indicated times. **e** Western blot detection of p-PDCD4 in HEK293 cells that were transfected with Flag-Atg7 or control vector incubating with Bafilomycin A1 (20 nM) for 12 h, then treated or not with 2DG (5 mM) and oligomycin (2.5 μM) stimulation for four hours. **f** In vivo ubiquitination assay in HEK293 Atg7-KO cells or Atg7-WT cells. **g** In vivo ubiquitination assay of HEK293 cells transfected with GFP-Atg7 or GFP-Atg7^C571S^ and the indicated plasmids. **h** In vivo ubiquitination assay of HEK293 cells transfected with GFP-Atg7 after 2DG (5 mM) and oligomycin (2.5 μM) treatment for four hours.

Since Atg7 regulates PDCD4 protein levels, it is anticipated to play a crucial role in PDCD4 ubiquitylation and degradation. To investigate the mechanism of Atg7-induced degradation of PDCD4, cycloheximide (CHX) was used to inhibit protein synthesis and MG132 was used to inhibit the ubiquitin-proteasomal pathway. Atg7 knockout was found to extend PDCD4 protein half-life (Fig. 2a, Supplementary Fig. 2a), and Atg7 overexpression accelerated the degradation rate of PDCD4 (Fig. 2b, Supplementary Fig. 2b). Thus, it can be concluded that Atg7 promotes PDCD4 degradation. Furthermore, the proteasome inhibitor MG132 was added to the cultured cells to investigate the degradation mechanism of PDCD4. The SDS-PAGE images revealed an increase in PDCD4 levels in Atg7 knockout cells (Fig. 2c, Supplementary Fig. 2c), while Atg7 overexpression resulted in reduced accumulation of PDCD4 (Fig. 2d, Supplementary Fig. 2d). To further research, we used Bafilomycin A1 (Fig. 2e) and CQ (Supplementary Fig. 2e) to inhibit autophagy and found that the ability of Atg7 to downregulate PDCD4 is independent of autophagy. These results suggest that Atg7 promotes the proteasomal degradation pathway of PDCD4. To verify the above findings, the ubiquitination level of PDCD4 was detected. The SDS-PAGE results showed that Atg7 knockout attenuated the ubiquitination of PDCD4 (Fig. 2f, Supplementary Fig. 2f). In addition, Atg7 wild type (Atg7-WT) and E1-like inactivated plasmid Atg7^C571S^ were used to elucidate the mechanism of Atg7-promoted PDCD4 ubiquitination. The findings indicated Atg7-WT and Atg7^C571S^ could enhance PDCD4 ubiquitination levels (Fig. 2g, Supplementary Fig. 2g), thus suggesting that Atg7 does not act as an E1 ubiquitin-activating enzyme of PDCD4. Thereafter, the ubiquitination level of PDCD4 under ATP depletion was estimated. The results imply the attenuated ubiquitination of PDCD4 led to PDCD4 protein accumulation, while Atg7 overexpression counterbalanced the reduced ubiquitination of PDCD4 (Fig. 2h, Supplementary Fig. 2h, i). Comprehensively, these findings indicated that the increase of PDCD4 due to ATP depletion is the result of PDCD4 accumulation owing to reduced ubiquitination.

### ATP sensing by Atg7 promotes PDCD4 phosphorylation at Ser67

**Fig. 3:**
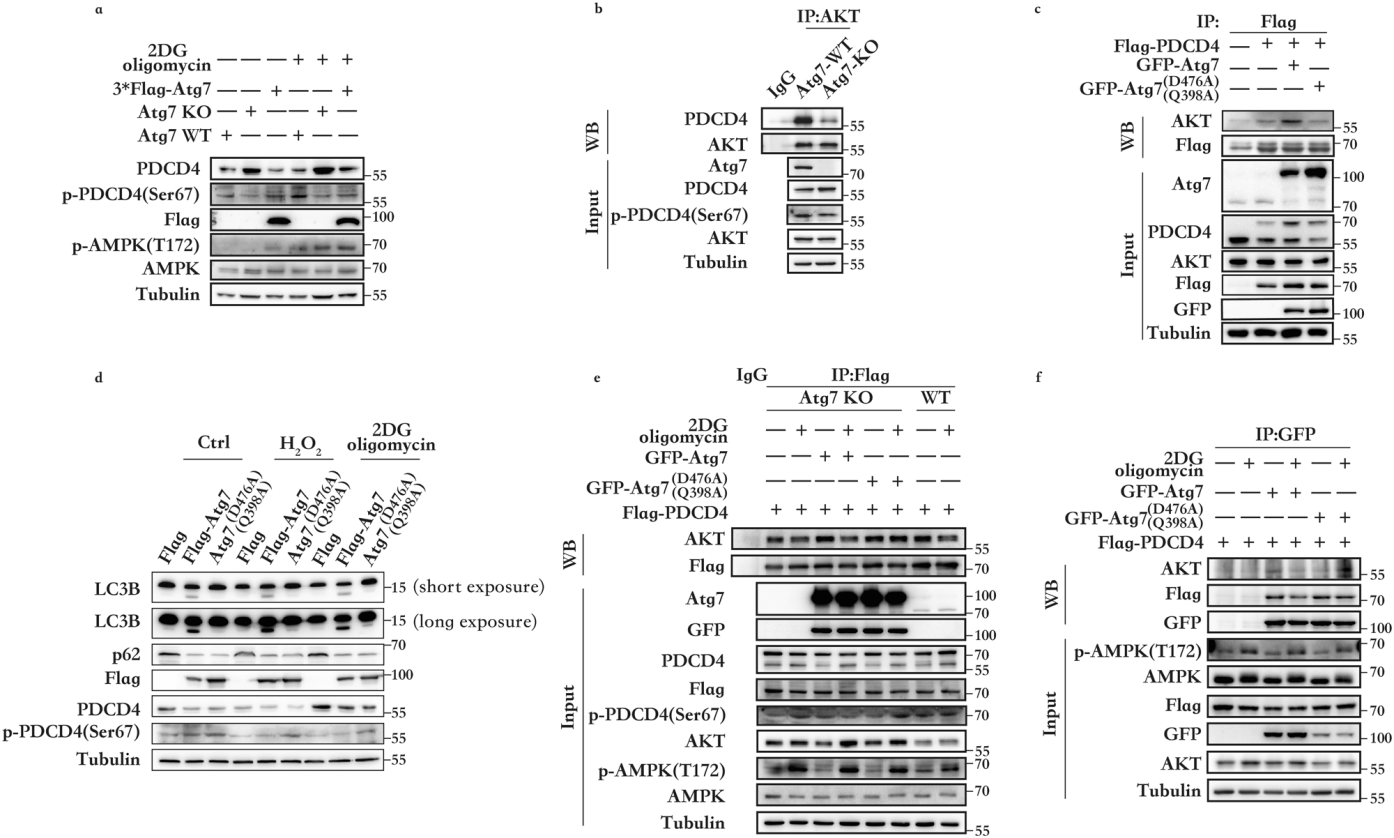
Atg7 senses ATP levels and promotes PDCD4 phosphorylation at Ser67. **a** Western blot detection of PDCD4 and p-PDCD4 Ser67 in HEK293 Atg7-WT, Atg7-KO, and HEK293 Atg7-KO cells transfected with Flag-Atg7 with or without 2DG (5 mM) and oligomycin (2.5 μM) stimulation for four hours. **b** Interaction between endogenous AKT and PDCD4 in HEK293 Atg7-KO and Atg7-WT cells. **c** HEK293 cells were transfected with GFP-Atg7, GFP-Atg7^D476AQ398A^, or control vector and the indicated plasmids. Co-immunoprecipitation assay was performed to detect the interaction between Flag and AKT. **d** HEK293 Atg7-KO cells were transfected with Flag-Atg7, Flag-Atg7^D476AQ398A^, or control vector. After pretreated with CQ (10 μM) in complete medium for 12 h and then treated or untreated with H_2_O_2_ (500 μM) or 2DG (5 mM) and oligomycin (2.5 μM) stimulation for four hours then collected for Western blot analysis. **e** HEK293 Atg7-KO cells were transfected with GFP-Atg7, GFP-Atg7^D476AQ398A^, or control vector and the indicated plasmids. Co-immunoprecipitation assay was performed to detect the interaction between Flag and AKT after 2DG (5 mM) and oligomycin (2.5 μM) treatment for four hours. **f** HEK293 cells were transfected with GFP-Atg7, GFP-Atg7^D476AQ398A^, or control vector and the indicated plasmids. Co-immunoprecipitation assay was performed to detect the interaction between GFP and AKT or Flag.

PDCD4 possesses two common phosphorylation sites: Ser67 and Ser457. Ser67 is phosphorylated by AKT and then degraded via the proteasome pathway, while phosphorylation of PDCD4 at Ser457 regulates its intracellular localization^25^. Atg7 was found not to directly ubiquitinate PDCD4 through its E1 activity. Since PDCD4 phosphorylation at Ser67 has been demonstrated to assume a critical role in Ubiquiti-proteasome degradation, it was tested whether Atg7 affects PDCD4 phosphorylation at Ser67. Accordingly, the phosphorylation of PDCD4 Ser67 in Atg7 knockout cells, Atg7-WT cells, and cells overexpressing Atg7 was studied. As shown in Fig. 3a, the deletion of Atg7, whether in the presence or absence of energy stress, downregulates the phosphorylation of PDCD4 at Ser67, while overexpression of Atg7 can upregulate the phosphorylation of PDCD4 at Ser67. It is to be noted that Atg7 is not a phosphorylated kinase and cannot directly cause PDCD4 phosphorylation at Ser67. Hence, the regulation of AKT-PDCD4 interactions by Atg7 was further explored. The experimental results indicated that in HEK293 Atg7 knockout cells, the binding of AKT to PDCD4 was attenuated (Fig. 3b).

Next, to explore the relationship between Atg7, AKT, and PDCD4, an adenosine domain inactive plasmid was constructed. Atg7 is an E1-like ubiquitin-activating enzyme and has a conserved adenosine structural domain like other E1s. Hence, the amino acid mutations at positions 476 and 398 were selected, and the feasibility of these mutations was verified by testing the ability of mutated Atg7 to activate LC3-I. Simultaneous mutations of D476 and Q398 most significantly attenuated the ability of Atg7 to activate LC3-I. Hence, Atg7^D476AQ398A^ was used for follow-up experiments. Further, the overexpression of Atg7-WT was found to promote the binding of PDCD4 to AKT but not Atg7^D476AQ398A^ (Fig. 3c).

We aim to demonstrate that Atg7^D476AQ398A^ do not affect Atg7’s ability to participate in autophagy. We will do so by using common autophagy-inducing stimuli such as oxidative stress (Fig. 3d), serum starvation (Supplementary Fig. 3a), glucose deprivation (Supplementary Fig. 3b), and HBSS (Supplementary Fig. 3c). Autophagic flux should remain functional. Atg7^D476AQ398A^ due to its loss of sensing ability, downregulates PDCD4 abnormally compared to the wild type under any stimulus.

In HEK293 Atg7-KO cells, we observed that reconstitution with wild-type Atg7 results in a reduction in the binding of the AKT to PDCD4 when compared to WT cells. However, reconstitution with Atg7^D476AQ398A^ shows no change in AKT binding to PDCD4 when compared to KO cells (Fig. 3e). In response to ATP depletion, AKT should weaken its binding to PDCD4, leading to PDCD4 accumulation. Reconstitution with Atg7^D476AQ398A^ just like Atg7 knock-out cells cannot achieve the change in AKT-PDCD4 binding observed in wild-type cells, indicating that Atg7 acts as an ‘ATP sensor’ in the AKT-PDCD4 complex. To validate the above conclusions, we observed howAtg7^D476AQ398A^ operates in HEK293 Atg7-KO cells with or without ATP. In the absence of ATP, Atg7^D476AQ398A^ results in increased binding of PDCD4 to AKT compared to wild-type Atg7 (Fig. 3f), this is the reason why Atg7^D476AQ398A^ downregulates PDCD4 abnormally.

Combinedly, Atg7 attenuates AKT phosphorylation of PDCD4 by sensing intracellular ATP changes, thereby reducing PDCD4 phosphorylation level at Ser67, leading to a reduction in PDCD4 ubiquitin proteasomal degradation.

### Atg7-PDCD4 pathway saves energy to reduce apoptosis

**Fig. 4:**
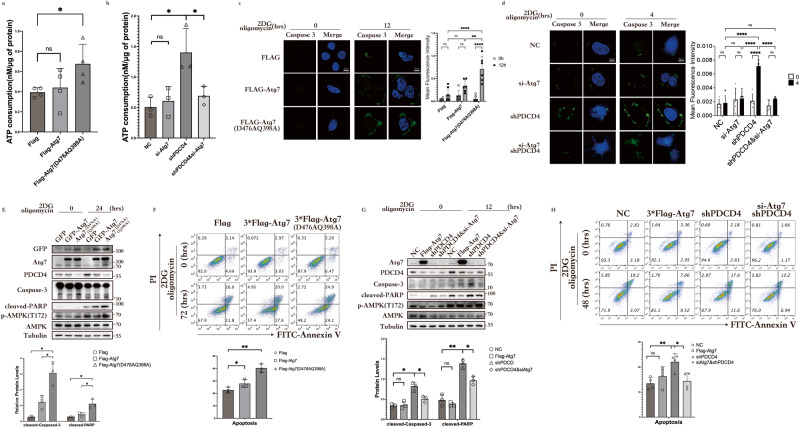
Atg7-PDCD4 pathway saves energy to reduce apoptosis. **a** HCT116 cells were transfected with Flag-Atg7, Flag-Atg7^D476AQ398A^ or control vector. Intracellular ATP levels were detected after 2DG (5 mM) and oligomycin (2.5 μM) treatment for four hours. Data represent the mean ± SD of four independent experiments (*n* = 4). *P* values were calculated by *t* test. *P < 0.05. **b** HCT116 cells or shPDCD4 cells were transfected with si-Atg7 or NC control. Intracellular ATP levels were detected after 2DG (5 mM) and oligomycin (2.5 μM) treatment for four hours. Data represent the mean ± SD of three independent experiments (*n* = 3). *P* values were calculated by *t* test. *P < 0.05. **c** HEK293 cells were transfected with Flag-Atg7, Flag-Atg7^D476AQ398A^ or control vector. Cells were labeled with fluorescent substrates for Caspase 3 (green) and mounted with DAPI. Then, they were visualized under a microscope. Data represent the mean ± SD of six independent experiments (*n* = 100 cells). *P* values were calculated by *t* test. *p < 0.05. Scale bar, 5 μm. **d** HCT116 cells or shPDCD4 cells were transfected with si-Atg7 or NC control. Cells were labeled with fluorescent substrates for Caspase 3 (green) and mounted with DAPI. Then, they were visualized under a microscope. Data represent the mean ± SD of five independent experiments (*n* = 100 cells). *P* values were calculated by *t* test. ****p < 0.0001. Scale bar, 5 μm. **e** HEK293 cells were transfected with Flag-Atg7, Flag-Atg7^D476AQ398A^ or control vector. After 2DG (5 mM) and oligomycin (2.5 μM) treatment for 24 h, a western blot was performed to detect PDCD4, Atg7, p-AMPKα Thr172, AMPKα, Caspase-3, and cleaved-PARP. The band intensities were quantified by gray values (*n* = 3). *P* values were calculated by *t* test. *P < 0.05. **f** HEK293 cells were transfected with Flag-Atg7, Flag-Atg7^D476AQ398A^ or control vector. After 2DG (5 mM) and oligomycin (2.5 μM) treatment for 72 h, the apoptosis was analyzed with FACS. Data represent the mean ± SD of three independent experiments (*n* = 3). *P* values were calculated by *t* test. *P < 0.05, **p < 0.005. **g** HCT116 cells were transfected with Flag-Atg7 or control. shPDCD4 cells were transfected with si-Atg7 or NC control. After 2DG (5 mM) and oligomycin (2.5 μM) treatment for 12 h, a western blot was performed to detect PDCD4, Atg7, p-AMPKα Thr172, AMPKα, Caspase-3, and cleaved-PARP. The band intensities were quantified by gray values (*n* = 3). *P* values were calculated by *t* test. *P < 0.05, **p < 0.005. **h** HCT116 cells were transfected with Flag-Atg7 or control. shPDCD4 cells were transfected with si-Atg7 or NC control. After 2DG (5 mM) and oligomycin (2.5 μM) treatment for 48 h, the apoptosis was analyzed with FACS. Data represent the mean ± SD of four independent experiments (*n* = 4). *P* values were calculated by *t* test. *P < 0.05, **p < 0.005.

To verify the capability of Atg7-PDCD4 pathway to save energy by inhibiting protein translation through PDCD4, the intracellular ATP content was assayed during energy depletion and Atg7^D476AQ398A^ overexpression was found to lead to an increase in ATP consumption (Fig. 4a). We believe the Atg7^D476AQ398A^ downregulates PDCD4 abnormally is the reason. Furthermore, we directly knocked down PDCD4 and observed a significant increase in ATP consumption in shPDCD4 cells. And the use of siRNAs against Atg7 in shPDCD4 cells resulted in slower ATP consumption (Fig. 4b).

We speculate that Atg7 has a dual role in the regulation of PDCD4: 1, It senses ATP and actively dissociates from the Atg7-PDCD4 complex. 2, It increase AKT-PDCD4 binding to downregulates PDCD4. In the absence of cellular stress, the binding of Atg7 to PDCD4 promotes the ubiquitination and degradation of PDCD4 through the ubiquitin-proteasome pathway. When Atg7 is artificially reduced, it is akin to directly reducing the binding of Atg7 to PDCD4, relieving Atg7’s downregulation of PDCD4 and leading to the accumulation of PDCD4 due to an increase in residual ATP. The excessive use of energy is speculated to activate apoptosis during the state of energy depletion. As reported, the loss of PDCD4 increased procaspase-3 expression, leading to its activation and PARP cleavage and sensitized the cells to apoptosis^5^. First, we observed the status of caspase-3 in the context of PDCD4 downregulation abnormally. The activity of caspase-3 in cells was assessed after 12 h of ATP depletion. The immunofluorescence studies revealed higher caspase-3 activity in Atg7^D476AQ398A^ cells than that in the Atg7-WT or control cells (Fig. 4c). Then, we measured ATP consumption in shPDCD4 cells after a four hours of ATP depletion. shPDCD4 cells exhibit higher caspase-3 activity. The use of siRNA against Atg7 in shPDCD4 cells reduced caspase-3 activity (Fig. 4d). After 24 h of ATP depletion, western blotting was performed. We found that the Atg7^D476AQ398A^ group has lower PDCD4 levels and higher cleaved-Caspase 3 expression (Fig. 4e). Flow cytometry apoptosis detection shown the same results, with the Atg7^D476AQ398A^ group exhibiting a higher apoptosis rate (Fig. 4f). These results suggest that the Atg7^D476AQ398A^ has lost the ability to sense ATP, preventing the dissociation of Atg7-PDCD4, thereby leading to abnormal downregulation of PDCD4. This phenomenon validates our hypothesis that Atg7 senses ATP in the regulation of Atg7-PDCD4.

To visually demonstrate the involvement of PDCD4 in apoptosis, we established a HCT116 shPDCD4 cell line. The silencing of PDCD4 exacerbated the apoptosis of HCT116 cells, and silencing Atg7 rescued the apoptosis caused by PDCD4 deletion (Fig. 4g). After 48 h of ATP depletion, the shPDCD4 group exhibited a higher apoptosis rate than that exhibited by the siAtg7shPDCD4 group (Fig. 4h). The inhibition of PDCD4 was relieved by artificially decreasing Atg7, and we observed the accumulation and upregulation of PDCD4 in the shPDCD4 group. This suggests that the attenuation of Atg7 indeed diminishes the ubiquitination degradation of PDCD4, leading to its accumulation, thereby alleviating apoptosis. This provides evidence for the involvement of the Atg7-PDCD4 pathway in apoptosis regulation.

All these results indicate that PDCD4, upregulated under ATP depletion, functions as an ATP-saving agent, effectively conserving energy to mitigate apoptosis. Furthermore, the Atg7-AKT-PDCD4 pathway, facilitated by Atg7’s ATP-sensing ability, initiates the upregulation of PDCD4 to balance cellular energy metabolism. It plays a crucial role in maintaining normal cellular ATP concentrations and assumes a critical role in cellular energy homeostasis.

## Discussion

A mechanism is discovered by which cells sense ATP consumption signals to inhibit protein translation and reduce energy consumption, thus safely surviving starvation. In energy-depleted states, Atg7 senses low ATP levels, and consequently, the Atg7-PDCD4 interaction is attenuated. In addition, PDCD4 expression is upregulated, which slows down cellular ATP consumption and enables the cell to avoid apoptosis.

PDCD4 is a tumor suppressor, and increasing evidence elucidates its detailed role in tumors. PDCD4 acts as a protein translation inhibitor, and thus inhibits tumor cell proliferation^26–28^, migration and invasion^4,29,30^. However, the role of PDCD4 in energy metabolism has not been clarified to date. Several researchers have reported that PDCD4 phosphorylation by AKT at Ser67 leads to the PDCD4 degradation that promotes G1 to S transition, thus regulating cell proliferation and growth^31^. Consistent with the previous studies, our results demonstrate that under normal conditions, Atg7 binds to PDCD4 to maintain low PDCD4 expression so that cells can maintain normal physiological activities. When cells respond to metabolic stress leading to ATP deprivation, Atg7 dissociates from PDCD4, allowing free PDCD4 to accumulate. The accumulation of PDCD4 leads to the inhibition of protein translation, and energy is saved. In their report, Y Bai. et al. also mentioned that PDCD4 can suppress the differentiation and self-renewal of adipocytes and PDCD4 deficiency increases energy expenditure^15^. The findings of the current study showed that in the early stage of energy depletion, as evidenced by AMPK activation, PDCD4 is upregulated due to the reduction of ubiquitinated degradation and upregulated PDCD4 responds to energy depletion. Therefore, a new perspective for research on PDCD4 is provided in this paper, which complements the function of PDCD4 from the perspective of maintaining energy homeostasis.

Previous studies on Atg7 have focused on its E1-like activity and ability to regulate autophagy^20^. Herein, the E1-like activity of Atg7 was found not to be directly involved in the regulation of PDCD4 ubiquitination. However, it promotes PDCD4 phosphorylation at Ser67 to indirectly promote PDCD4 degradation. Furthermore, the adenylated structural domain of Atg7 was explored based on X-ray crystallography, and the available structural domain data were used to mutate that domain to inactivate it. Since the adenylated structural domain of the Atg7 is homologous in the E1-like activating enzymes, the mutation site of Atg7 was chosen in reference to the related study of Uba3^32^. Our findings suggest that Atg7 can sense ATP levels, a function that is not dependent on autophagy. When AMPK activation was evidenced as a result of ATP deprivation, the presence or absence of Atg7 was found to alter the interaction between AKT and PDCD4. In addition, it was also revealed that Atg7 and Atg7^D476AQ398A^ exhibited varying abilities to promote AKT binding to PDCD4.

Our findings indicated that Atg7 overexpression could counteract the increase in PDCD4 during ATP depletion. For example, in Fig. 2G, Atg7 overexpression at four hours of ATP deprivation still upregulated PDCD4 ubiquitination compared to the control, and PDCD4 protein levels were lower than that in the control. This observation is probably because of a delay between transfection and ATP deprivation during the experiment. Atg7 overexpression is completed in the complete medium used for culture and on sensing there is no ATP deficiency at this time, Atg7 promotes the ubiquitination degradation of PDCD4.

Previous studies in our laboratory revealed that Atg7 inhibited the Warburg effect by suppressing the PKM2 phosphorylation^33^. The results from the current study show that Atg7 promotes PDCD4 phosphorylation at Ser67 to maintain normal physiological activity. Furthermore, PDCD4 inhibits anabolism to save energy in the absence of ATP. As mentioned in previous reports, Atg7 can function as a regulator of metabolism without relying on autophagy. Moreover, we propose that Atg7 can sense alterations in ATP levels, thus refining the role of Atg7 in metabolic homeostasis.

## Materials and methods

### Cell lines and cell culture

HEK293 was preserved by our laboratory and cultured in DMEM with 10% FBS. HCT116 was also preserved by our laboratory and cultured in IMDM with 10% FBS. All cells were grown at 37 °C in a 5% CO2 incubator.

### Plasmid constructions and viral infection

GFP-Atg7 and GFP-Atg7^D476AQ398A^ were constructed by PCR amplification and subcloned into pEGFP-C1 (CLONTECH Laboratories, Inc. 6084-1) vectors.

3*Flag-Atg7 and 3*Flag-PDCD4 were constructed by PCR amplification and subcloned into pcDNA3.0 (provided by Feng Li) vectors.

HA-Ubiquitin was gifted by Zhou tingting.

GST-PDCD4 and GST-Atg7 were constructed by PCR amplification and subcloned into pGEX-5x-1 (GE Healthcare, 28-9545-53) vectors.

The sequence of sgRNA for Atg7 was as follows^34^:

sgRNA1: AATAATGGCGGCAGCTACGG;

sgRNA2: AAAGCTGACACTATACTGG.

Atg7 and PDCD4 siRNA were purchased from RiboBio.

For lentiviral production and infection, control shRNA (shCtrl) lentivirus, and shRNA against PDCD4 (shPDCD4) were purchased from Shanghai GeneChem Company.

### Antibodies and reagents

The following antibodies were used for immunoprecipitation(IP) or immunoblots(IB): PDCD4 antibody (IP:1:200, IB:1:1000, Santa Cruz Biotechnology, sc-376430; Cell Signaling Technology, #9535); phospho-PDCD4(Ser67) antibody (IB: 1:1000, Abcam, ab73343; Sangon Biotech, D151427); Atg7 antibody (IP:1:200, IB:1:1000, Sigma, SAB1407006); AKT antibody (IP:1:200, IB:1:1000, Cell Signaling Technology, #4691); AMPKα (IB:1:1000, Cell Signaling Technology, #2532); Phospho-AMPKα(Thr172)(IB:1:1000, Cell Signaling Technology, #2531), Caspase3 antibody (IB:1:1000,Cell Signaling Technology, #9662); Cleaved PARP antibody (IB:1:1000, Cell Signaling Technology, #5625); Ubiquitin antibody (IB:1:1000, Cell Signaling Technology, #3933); HA-Tag antibody (IB:1:1000, Cell Signaling Technology, #3724); Flag antibody (IB:1:1000, Genomics Technology, SG4110-16); GFP antibody (IB:1:1000, GenScript, A01388-40); α-Tubulin antibody (IB:1:2000, Cell Signaling Technology, #2144); Anti-Flag Affinity Gel (B23102) was from Bimaker.; Protein A/G agarose (SC-2003)was from Santa Cruz.; CHX (S7418) and MG132 (S2619) were from Selleck.; jetPRIME was from Polyplus.

### Quantitative real-time PCR

RNA was isolated from cells using RNAiso Plus reagent (Takara, #9109), and then, reverse transcription was performed using a PrimeScript RT-PCR Kit (Takara, RR047A). Quantitative real-time PCR was performed using TB Green Premix *Ex Taq* II (Takara, RR820A) on Roche LightCycler 480 system. The data were normalized to *GAPDH* mRNA. The following primers were used for qPCR: 5′-TGGATTAACTG TGCCAACCA-3′ and 5′-TCTCAAATGCCCTTTCATC C-3′ for *PDCD4*^35^;

5′-TGCTATCCTGCCCTCTGTCTT-3′ and 5′-TGCCTCCTTTCTGGTTCTTTT-3′ for *Atg7*^36^;

5′-GTCTCCTCTGACTTCAACAGCG-3′ and 5′-GTCTCCTCTGACTTCAACAGC G-3′ for *GAPDH*^35^.

### Generation of ATG7 CRISPR/Cas9 KO cells

To establish a stable Atg7 Cas-9 knockout HCT-116 and HEK293 cell line, sgRNA was annealed and inserted into Cas-9 vacant. Then, the successfully sequenced Atg7 Cas-9 plasmid was transfected into HCT-116 /HEK293 cells. After 48 h culture, puromycin (20 µg/mL) was added to the screen for 72 h. Again after 72 h, the cells were treated with puromycin (2 µg/mL) in order to ensure growth under single cell conditions. The Atg7 KO colonies were expanded and confirmed by PCR analysis and western blot.

### Immunoblots and immunoprecipitation

Cells were lysed for 30 min using IP lysis buffer (25 mM Tris, pH 7.6, 150 mM NaCl, 1% Nonidet P-40, 1 mM EDTA) and protease inhibitors, and then centrifuged at 13,500 rpm for 20 min. One milligram protein and 5 µL antibody were incubated for 4 h before 20 µL protein A/G agarose beads were added and kept overnight. Then, the beads were washed thrice using PBS and tested by western blot.

### GST pull-down assays

GST-Atg7 or GST-PDCD4 fusion proteins were purified from BL21 competent cells using GST sepharose beads (GE Healthcare, 17-0756-01). 3*Flag-Atg7 or 3*Flag-PDCD4 were transcribed using T7-TNT kits (Promega, L1170) according to the manufacturer’s standard procedures. Then, GST protein or GST-Atg7 fusion protein was incubated with 3*Flag-PDCD4 for four hours. Thereafter, the beads were washed thrice using PBS followed by a western blot. For in vivo GST-PDCD4 and 3*Flag-Atg7 interaction, GST protein or GST-PDCD4 fusion protein was incubated with 3*Flag-Atg7 for four hours. Thereafter, the beads were washed thrice using PBS followed by a western blot.

### Flow cytometry assay

For cell apoptosis assay, cells were transfected with siRNA. After 24 h, the experimental groups were subjected to ATP depletion for 48 h. Then, the frequency of apoptotic cells was tested using an Annexin V-FITC/PI kit (KeyGENBioTECH, KGA108) following the manufacturer’s standard procedures.

### Caspase-3 activity assay

HCT116 cells were grown on glass bottom dishes and incubated for 30 min at room temperature with 5 μM substrates of Caspases 3 (Beyotime, C1168M), then fixed with 4% paraformaldehyde for 1 h, and eventually mounted with DAPI (Sigma, 32670). The images were obtained using a 60X oil lens objective on an inverted fluorescence microscope (Nikon, Ti-E, DS-Ri2, NY USA).

### Measurement of cellular ATP concentration

Cells were lysed to measure the ATP concentration following the manufacturer’s protocol of the ATP Determination Kit (Beyotime, S0026).

### Immunofluorescence assay

The cells were transfected with plasmids and then inoculated onto coverslips. Next, these cells were washed thrice with ice-cold PBS, fixed with 4% paraformaldehyde for 1 h, and then permeabilized with 0.5% Triton X-100 for 20 min. Thereafter, blocking was performed with 20% goat serum in 0.5% Triton X-100. Fixed cells were incubated overnight at 4 °C with the corresponding primary antibody. Coverslips were then washed in 0.5% Triton X-100 and stained for 60 min with Alexa Fluor 488- /Alexa Fluor 594-conjugated secondary antibody. Then, the coverslips were rewashed with PBS, then mounted with DAPI. For the co-localization assay, images were obtained using a 60X oil lens objective on an inverted (GE Healthcare, DeltaVision OMX 3D-SIM).

### Statistics and reproducibility

The results were expressed as mean ± standard deviation (Mean ± SD) from at least three independent experiments. The statistical analysis was performed using GraphPad Prism Version 9.4.1 software. The differences between multiple groups of data were compared by applying two-way ANOVA, and differences between two groups of data were compared by applying the Student t-test. A final p-value less than 0.05 indicated a statistical significance.

### Reporting summary

Further information on research design is available in the Nature Portfolio Reporting Summary linked to this article.

### Supplementary information

Supplementary Information

Description of Additional Supplementary Files

Supplementary Data 1

Reporting Summary

## Data Availability

The numerical source data underlying all graphs can be found in Supplementary Data 1. The flow cytometry gating information can be found in Supplementary Fig. 4. Uncropped blots can be found in Supplementary Fig. 5. Additional datasets analysis generated during this study are available on reasonable request.
