# Supplementary material for: Atg7 senses ATP levels and regulates AKT1-PDCD4 phosphorylation-ubiquitination axis to promote survival during metabolic stress
Source: Commun Biol. 2023 Dec 11;6:1252. doi: 10.1038/s42003-023-05656-7 (PMC10713595; doi:10.1038/s42003-023-05656-7)

Supplementary Figure 1. Atg7 interacts with PDCD4 and negatively regulates PDCD4 protein levels

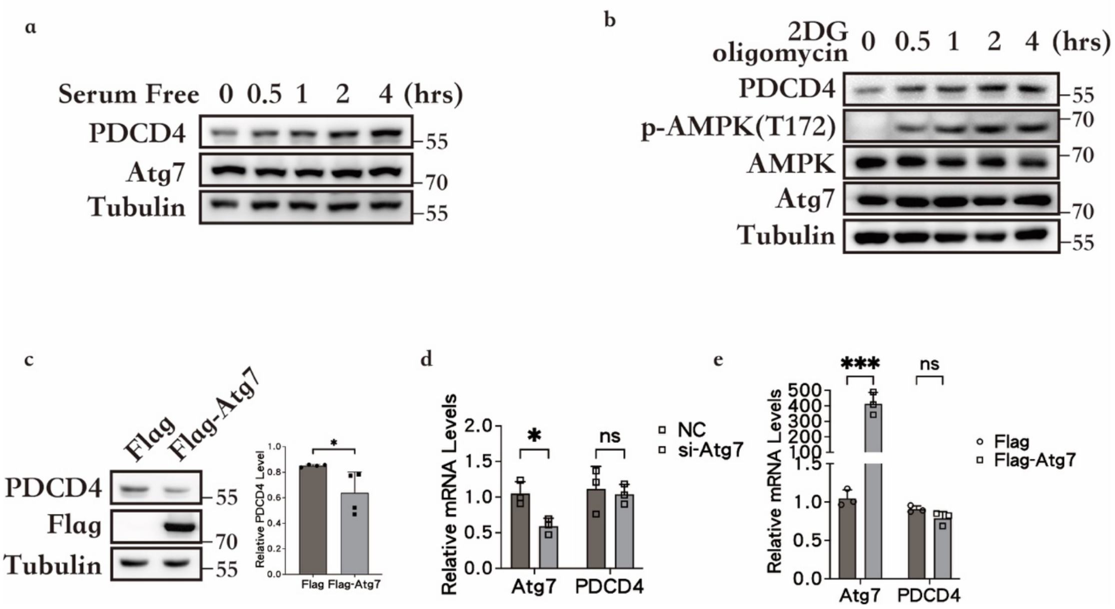

a. Western blot detection of PDCD4 and Atg7 in HCT116 cells in normal medium or serum-free medium for the indicated times.

b. Western blot detection of PDCD4, Atg7, p-AMPKα Thr172, and AMPKα in HCT116 cells in normal medium or after 2DG (5 mM) and oligomycin (2.5 μM) treatment for the indicated times.

c. PDCD4 expression levels were decreased in HCT116 cells transfected with Flag-Atg7 compared with control cells. Data represent the mean ± SD of three independent experiments. P values were calculated by t-test. *P<0.05

d. HEK293 cells were transfected with si-Atg7 or NC control, followed by qRT-PCR to determine mRNA expression. Data represent the mean ± SD of three independent experiments. P values were calculated by t-test. *P<0.05.

e. HEK293 cells were transfected with Flag-Atg7 or vector control, followed by qRT-PCR to determine mRNA expression. Data represent the mean ± SD of three independent experiments. P values were calculated by t-test. ***p < 0.001.

Supplementary Figure 2. Atg7 degrades PDCD4 through the ubiquitin-proteasomal pathway

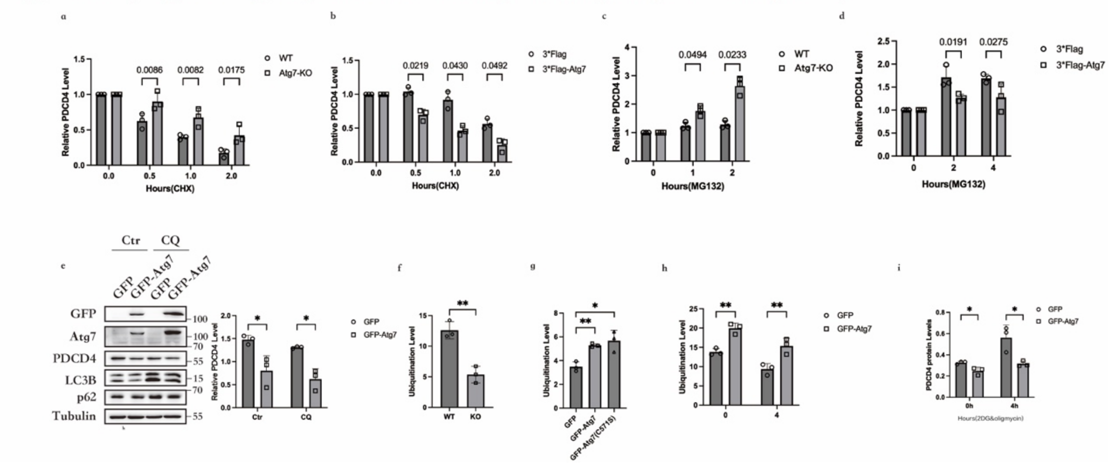

a. The band intensities were quantified by gray values. P values were calculated by two-way ANOVA.

b. The band intensities were quantified by gray values. P values were calculated by two-way ANOVA.

c. The band intensities were quantified by gray values. P values were calculated by two-way ANOVA.

d. The band intensities were quantified by gray values. P values were calculated by two-way ANOVA.

e. HEK293 cells were transfected with GFP-Atg7 or control vector. After pretreated with CQ (10μM) in complete medium for 12 hours then collected for Western blot analysis. The band intensities were quantified by gray values. P values were calculated by two-way ANOVA. *P<0.05

f. The band intensities were quantified by gray values. P values were calculated by t-test. **P<0.01

g. The band intensities were quantified by gray values. P values were calculated by t-test. *P<0.05, **P<0.01

h,i. The band intensities were quantified by gray values. P values were calculated by two-way ANOVA. *P<0.05, **P<0.01

Supplementary Figure 3. Atg7 senses ATP levels and promotes PDCD4 phosphorylation at Ser67

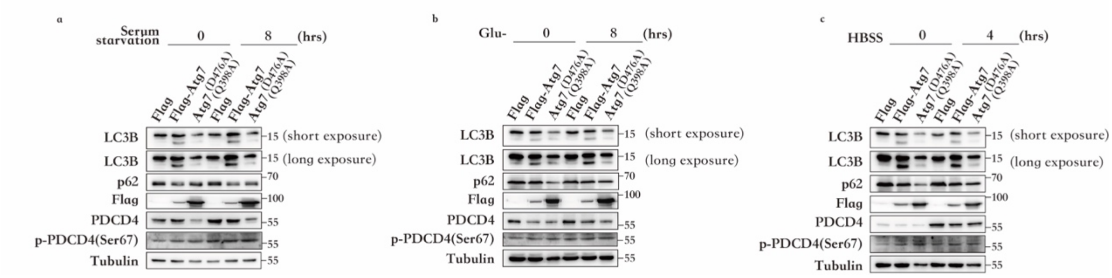

a. Atg7-KO cells were transfected with Flag-Atg7, Flag-Atg7^{D476AQ398A}, or control vector. Then treated or untreated with serum starvation stimulation for eight hours then collected for Western blot analysis.

b. Atg7-KO cells were transfected with Flag-Atg7, Flag-Atg7^{D476AQ398A}, or control vector. Then treated or untreated with glucose starvation stimulation for eight hours then collected for Western blot analysis.

c. Atg7-KO cells were transfected with Flag-Atg7, Flag-Atg7^{D476AQ398A}, or control vector. Then treated or untreated with HBSS starvation stimulation for four hours then collected for Western blot analysis.

Supplementary Figure 4. Gating strategy

Figure 4f

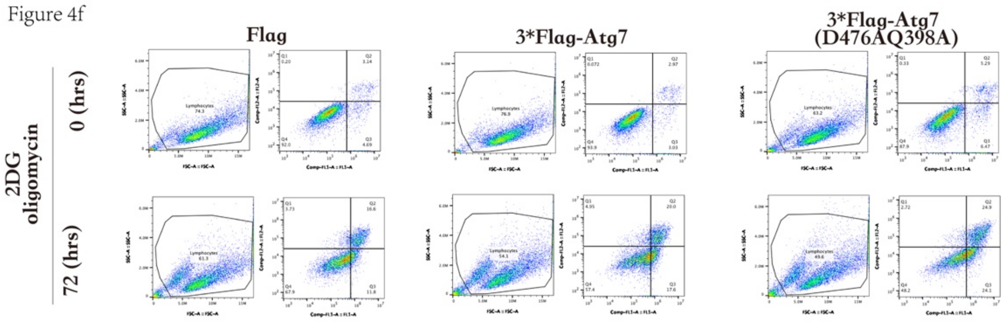

Figure 4h

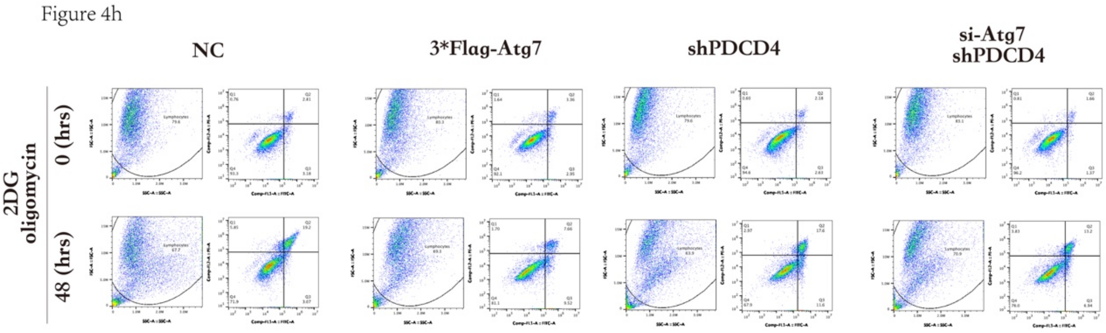

Supplementary Figure 5. Uncropped blots

Figure 1a

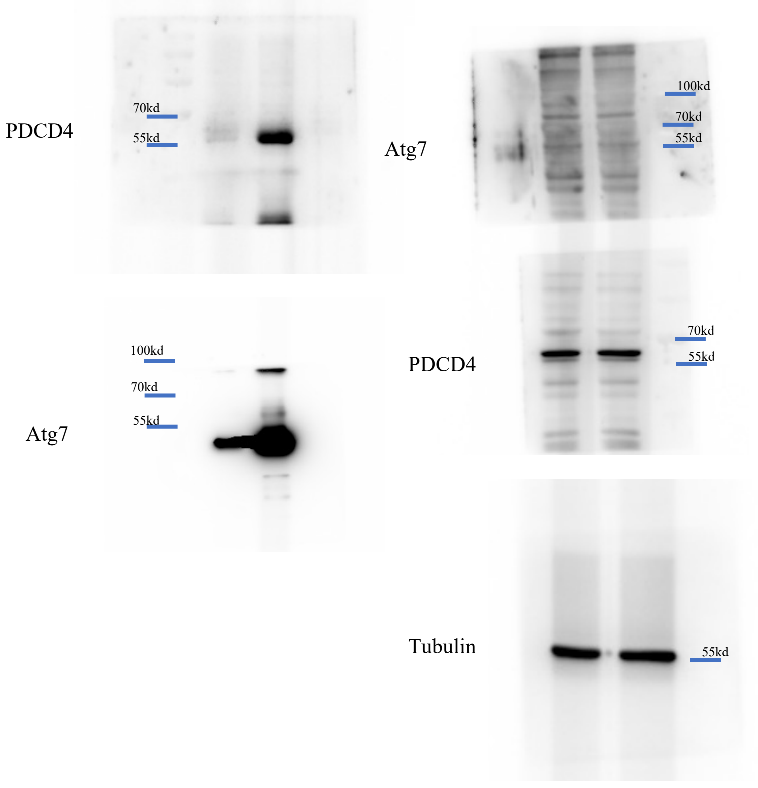

Figure 1b

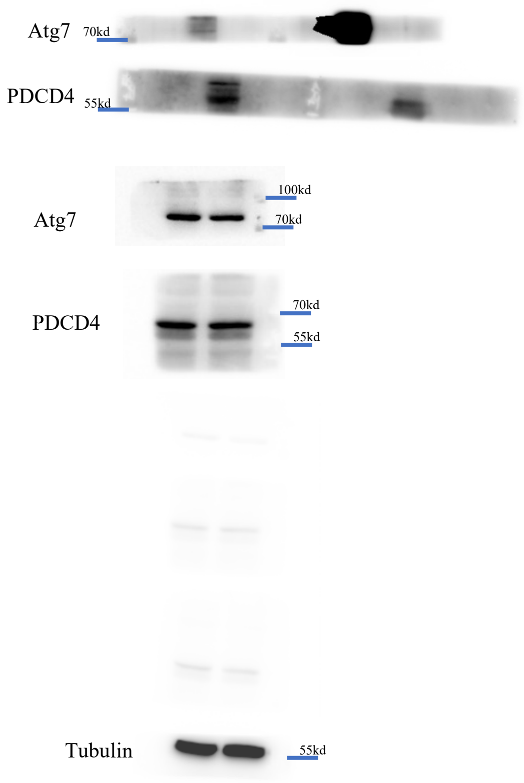

Figure 1c

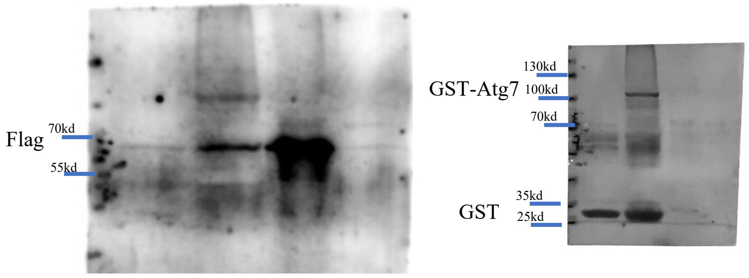

Figure 1d

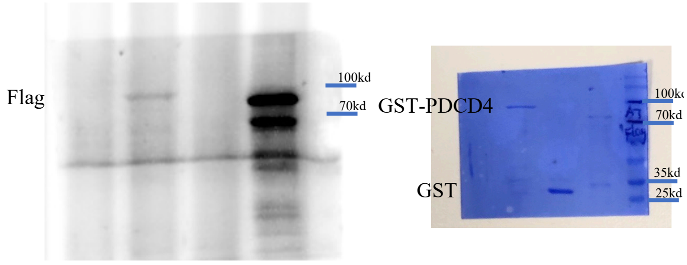

Figure 1f

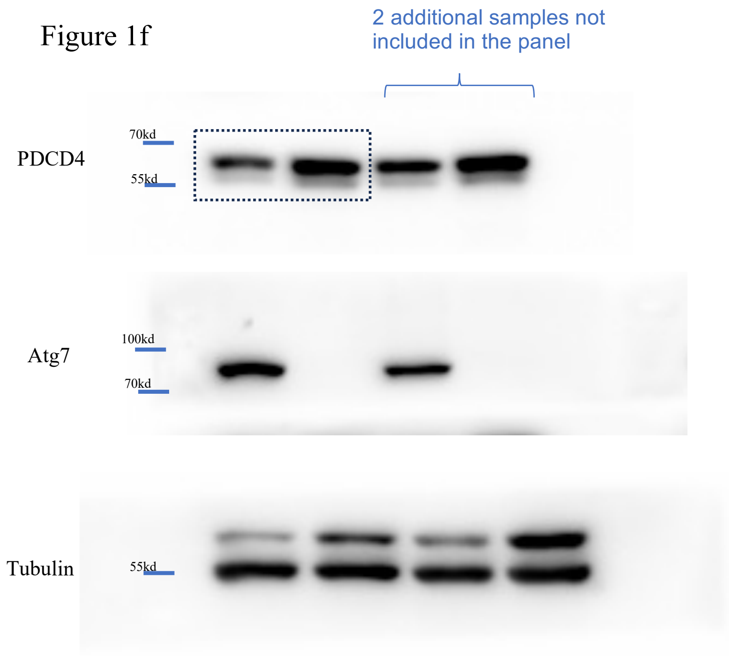

Figure 1g

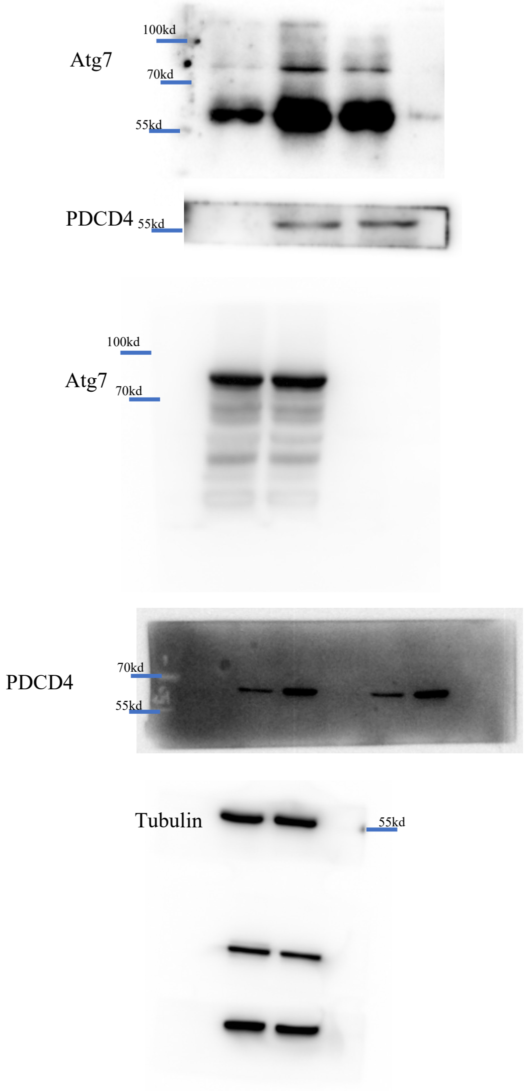

Figure 1h

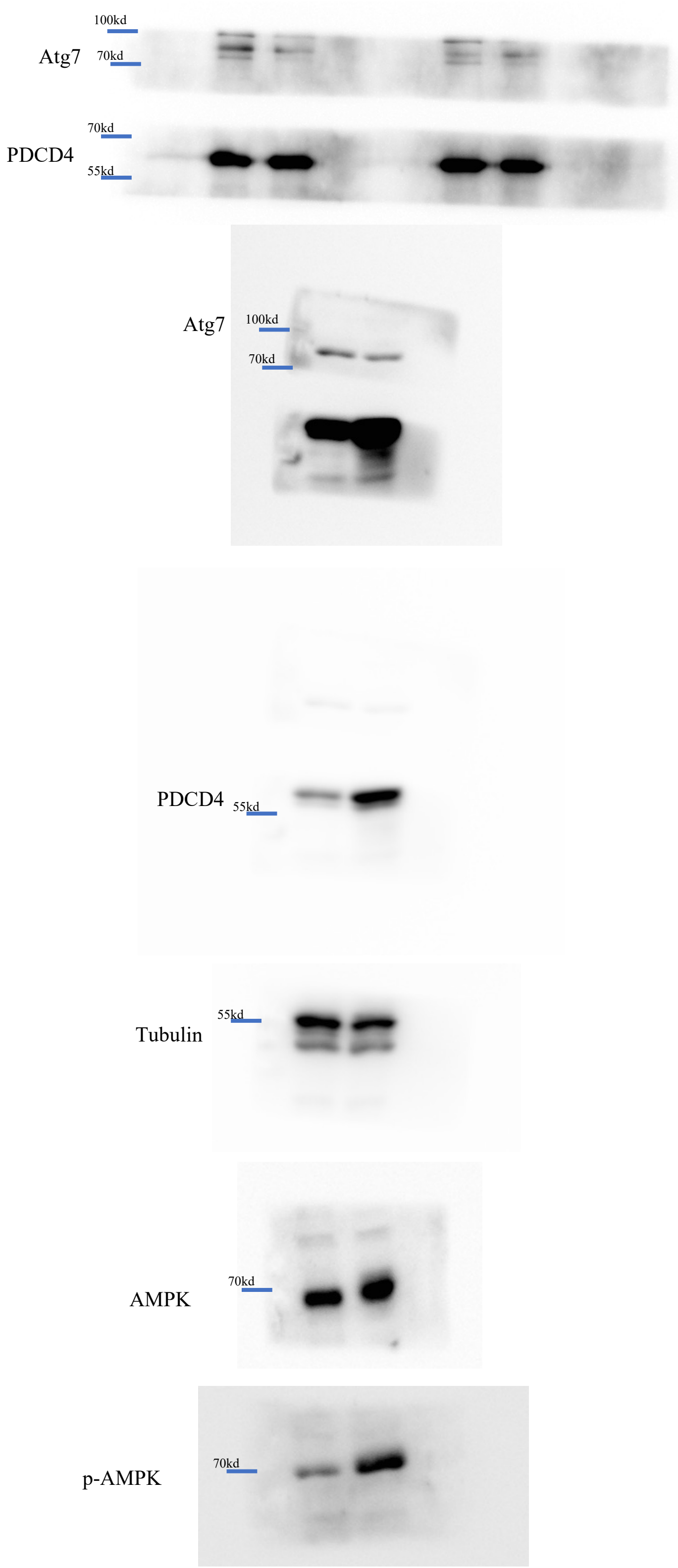

Figure 2a

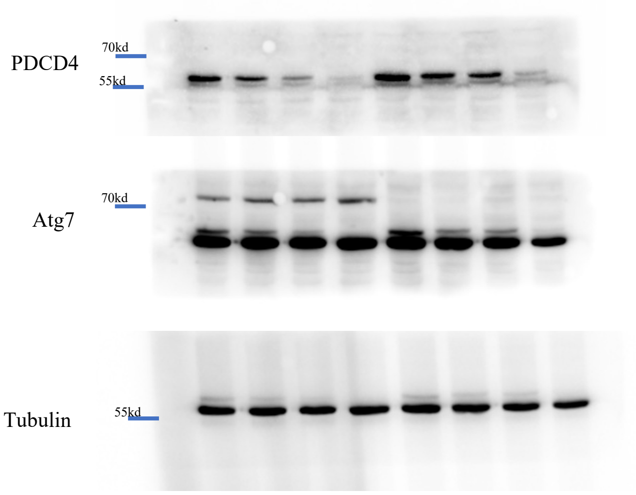

Figure 2b

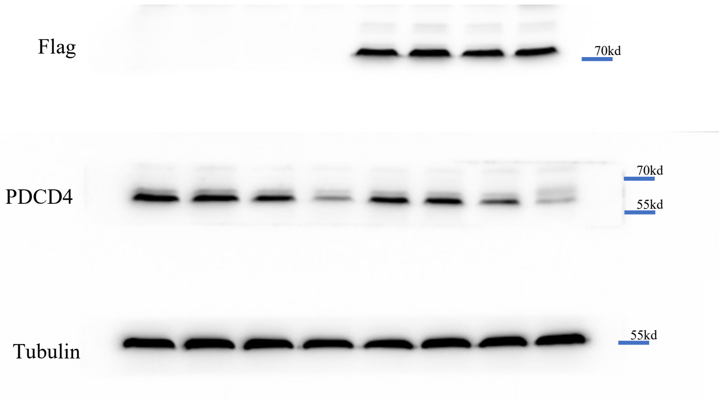

Figure 2c

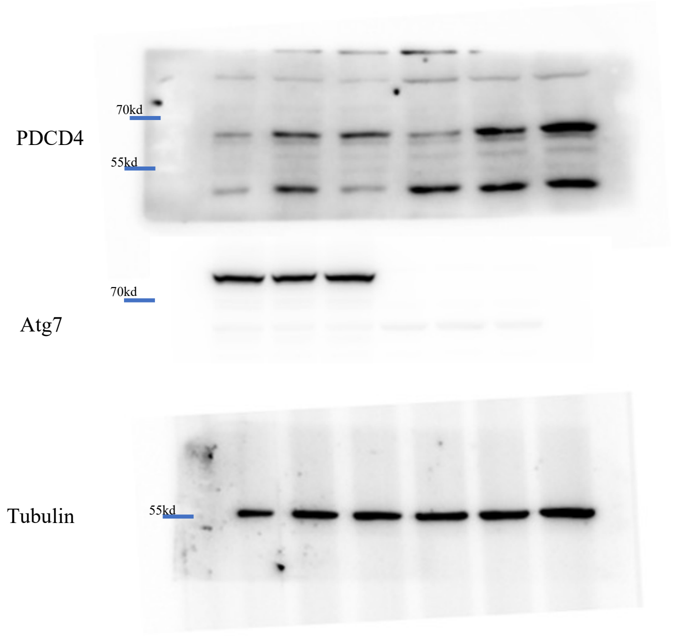

Figure 2d

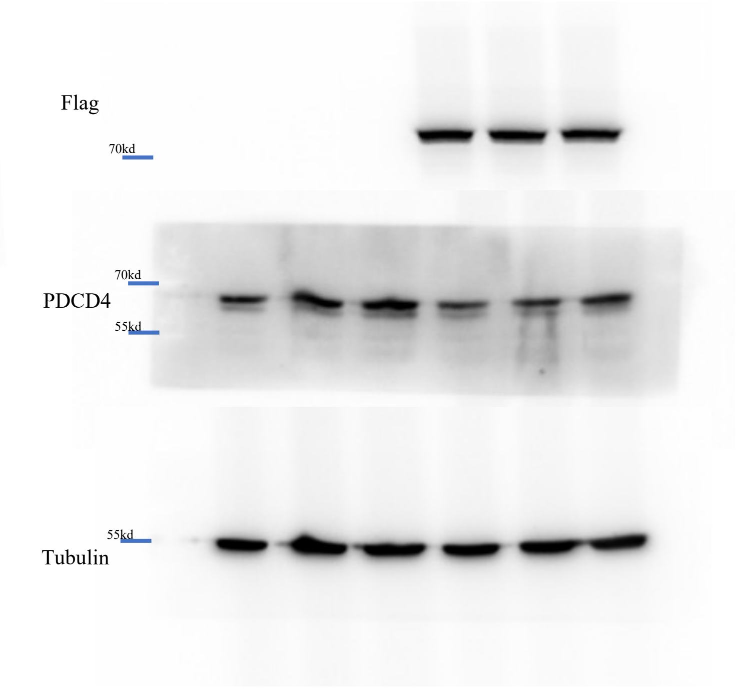

Figure 2e

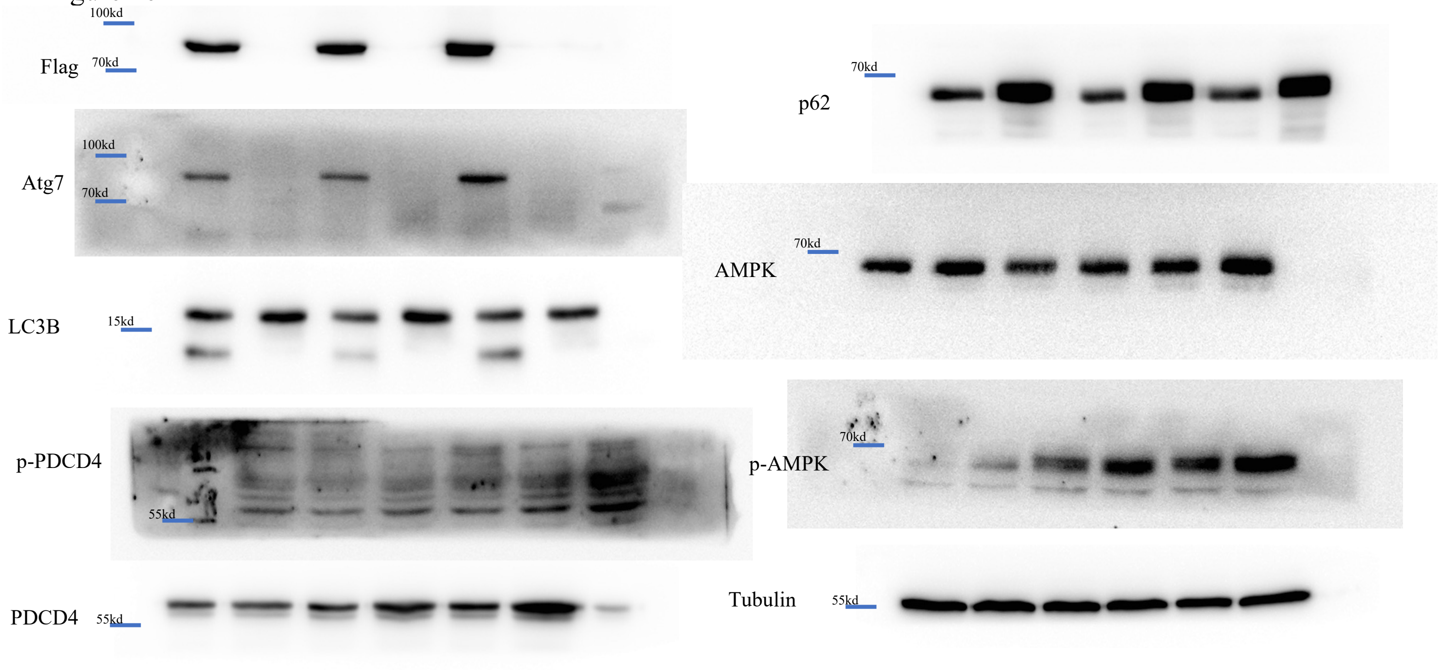

Figure 2f

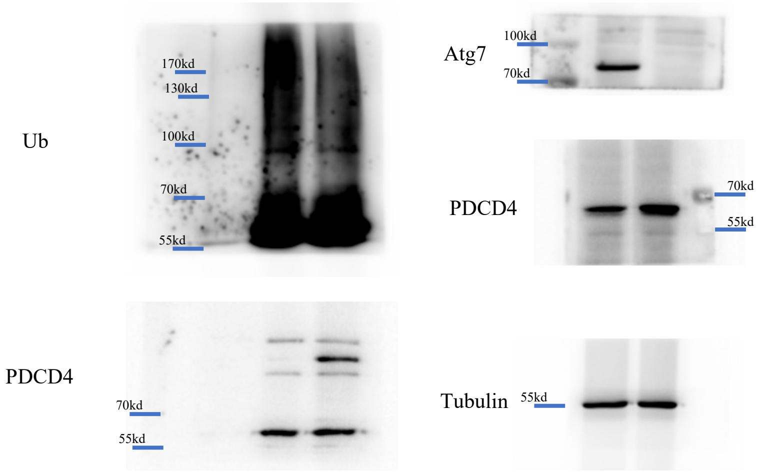

Figure 2g

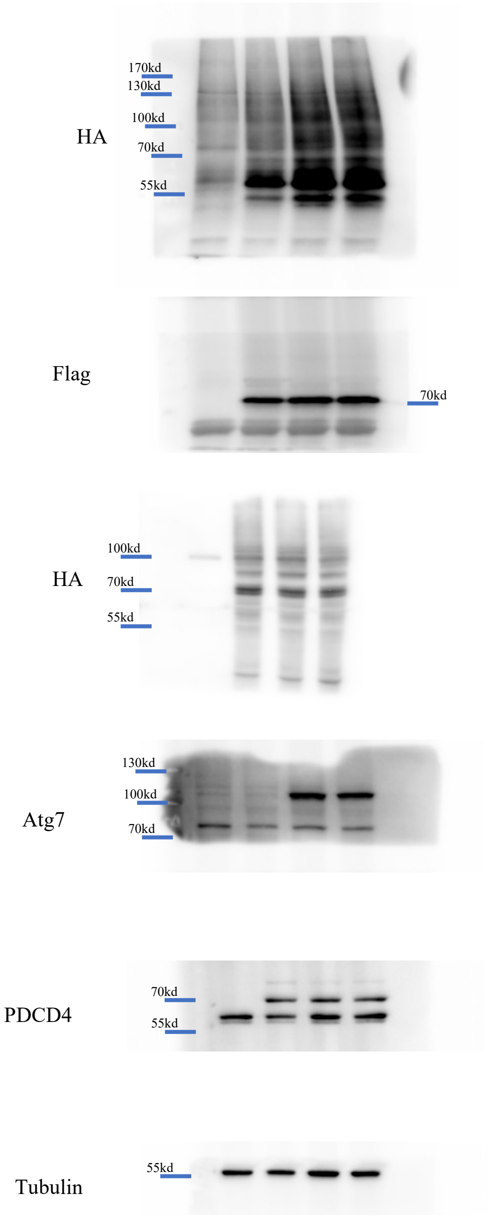

Figure 2h

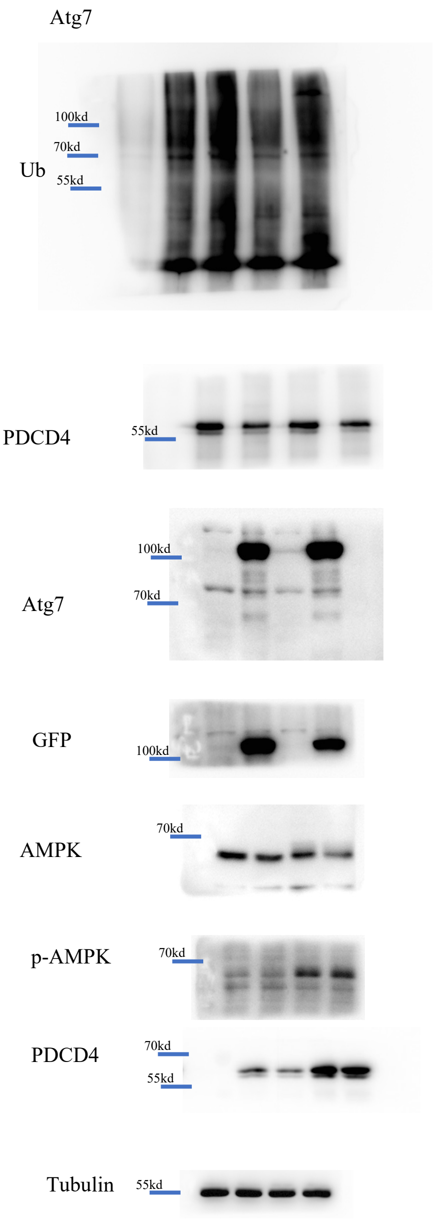

Figure 3a

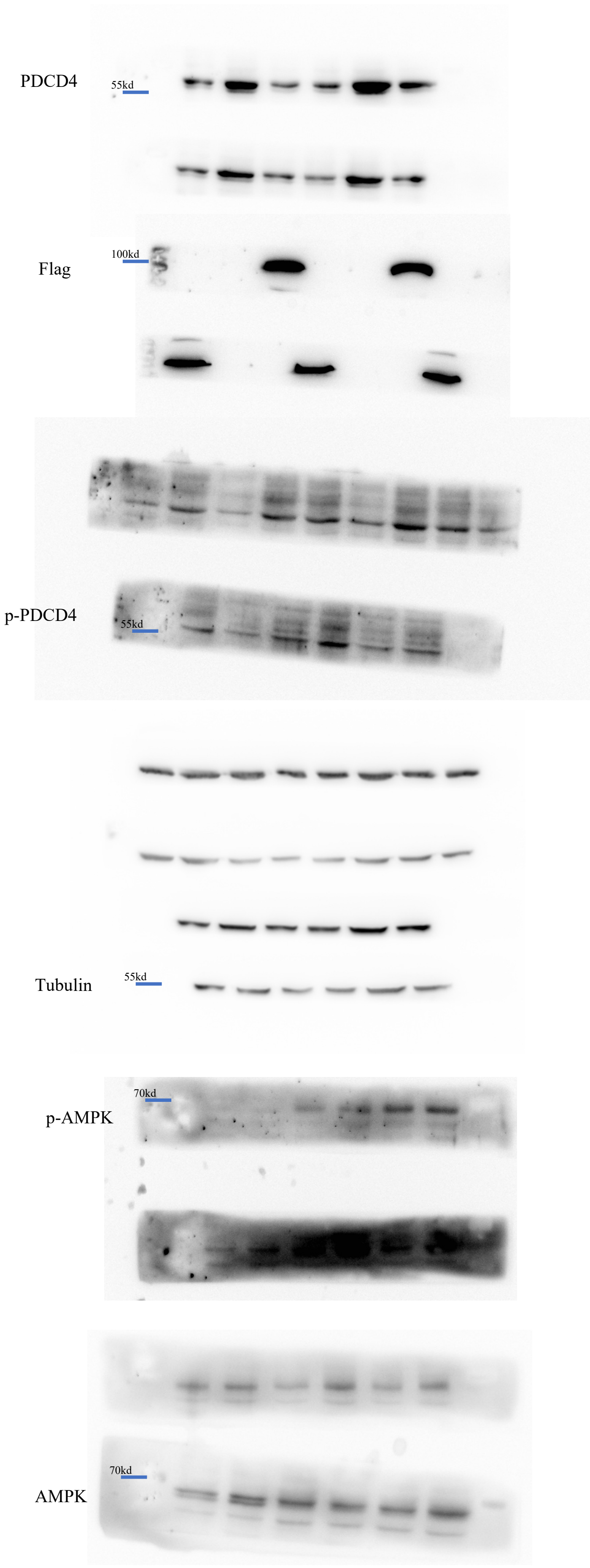

Figure 3b

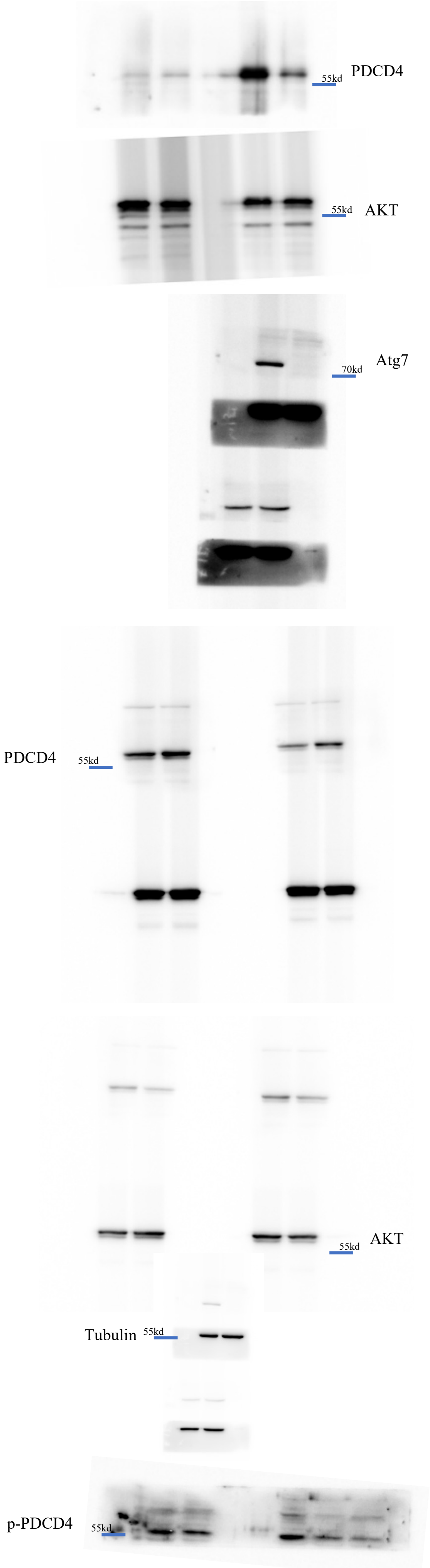

Figure 3c

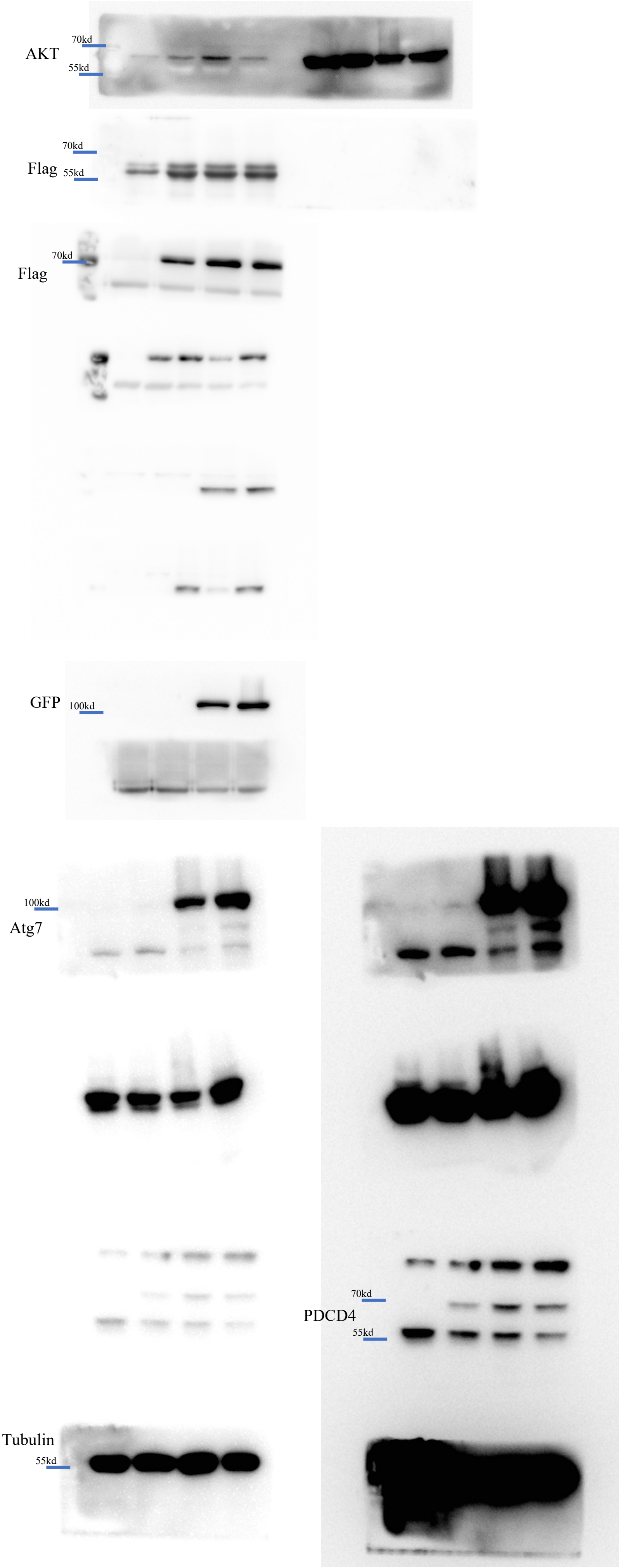

Figure 3d

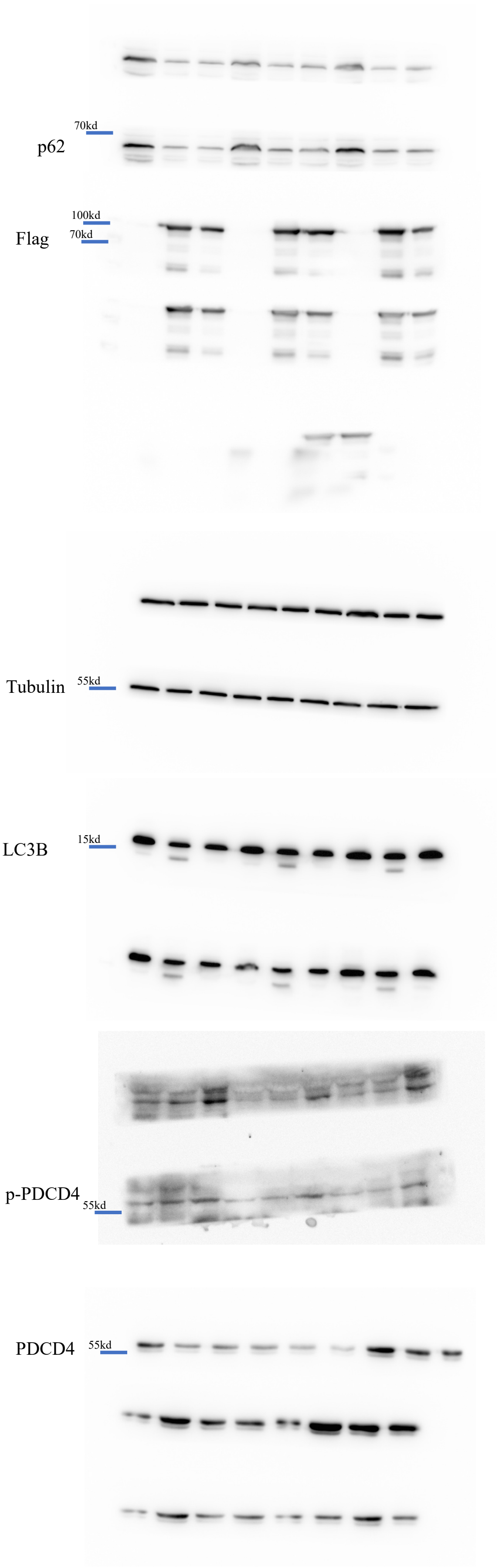

Figure 3e

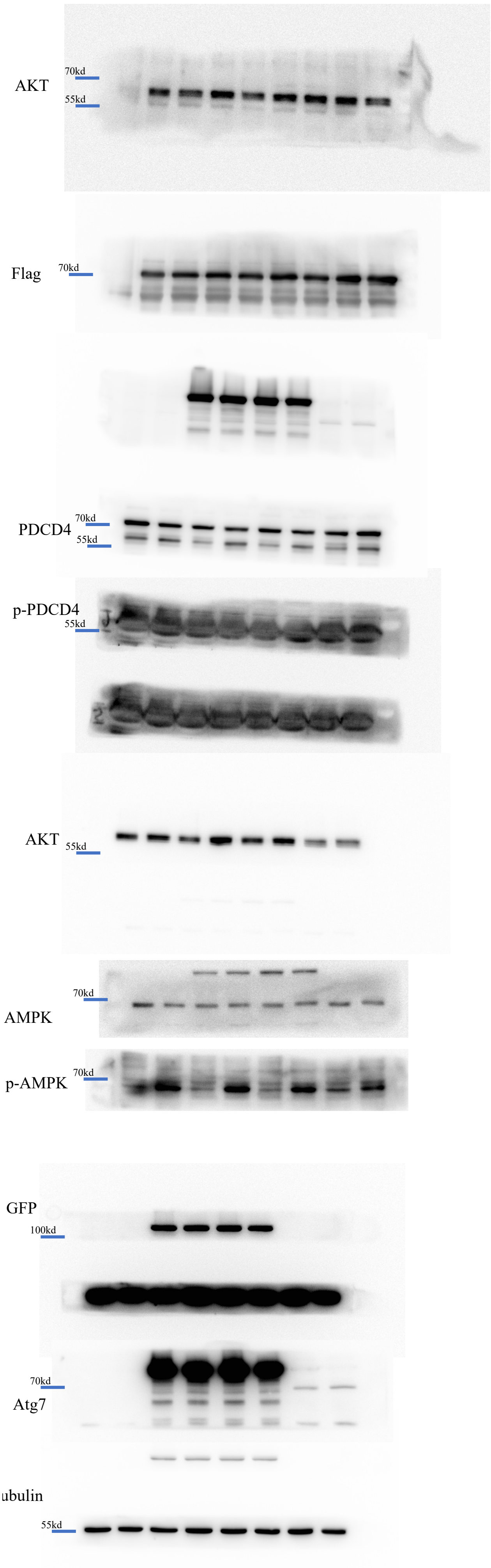

Figure 3f

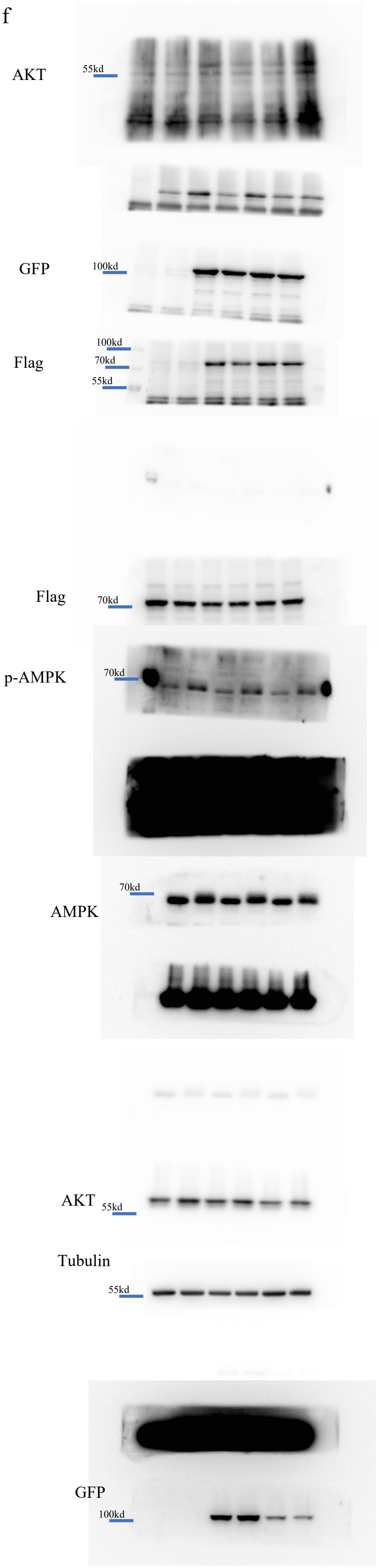

Figure 4e

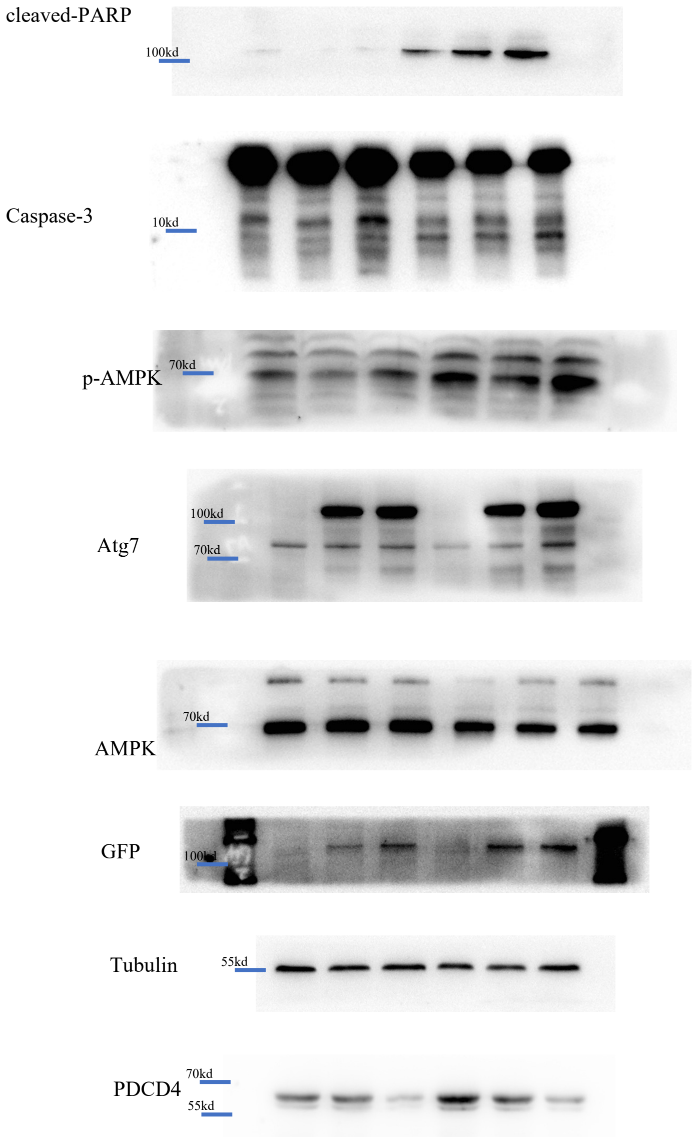

Figure 4g

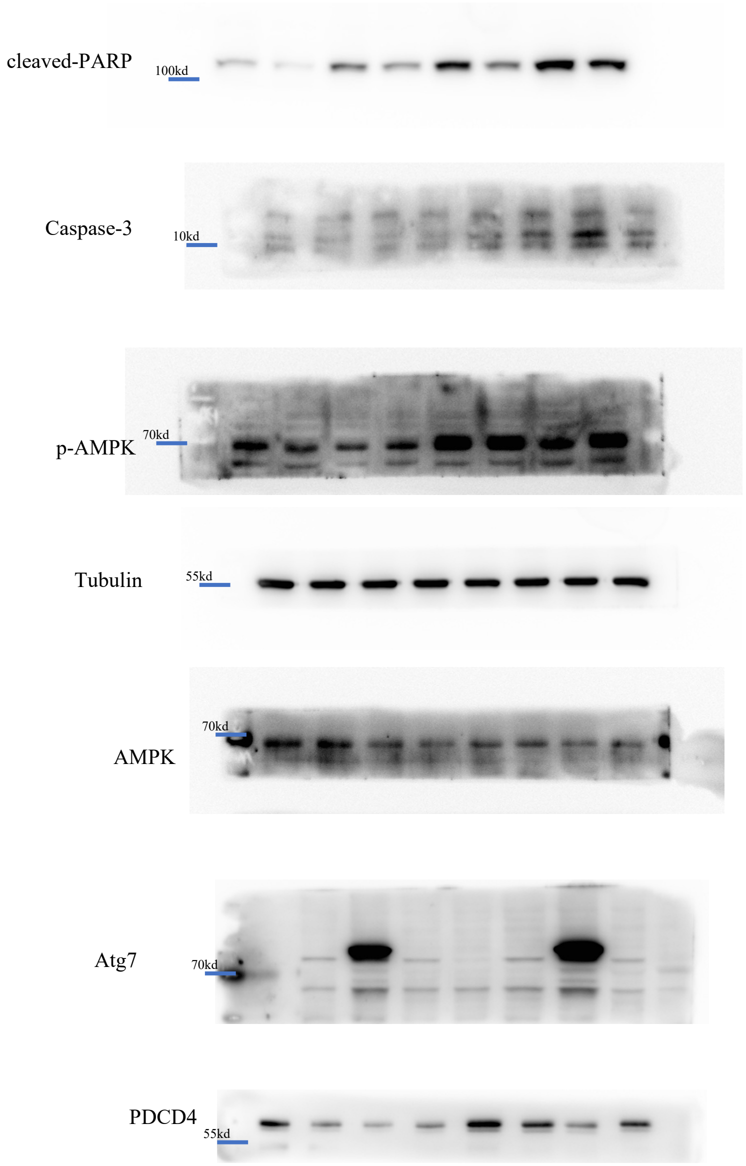

Supplementary Figure 1a

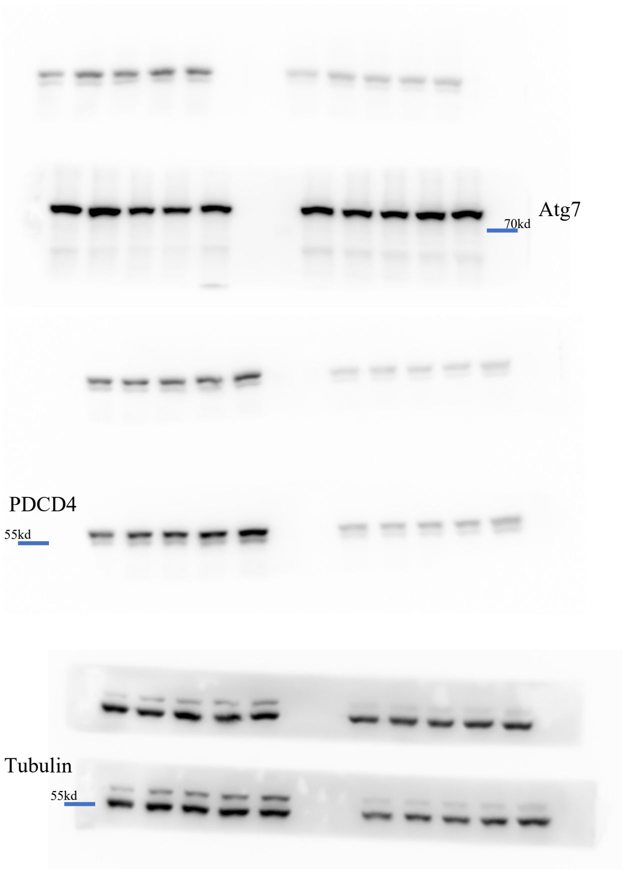

Supplementary Figure 1b

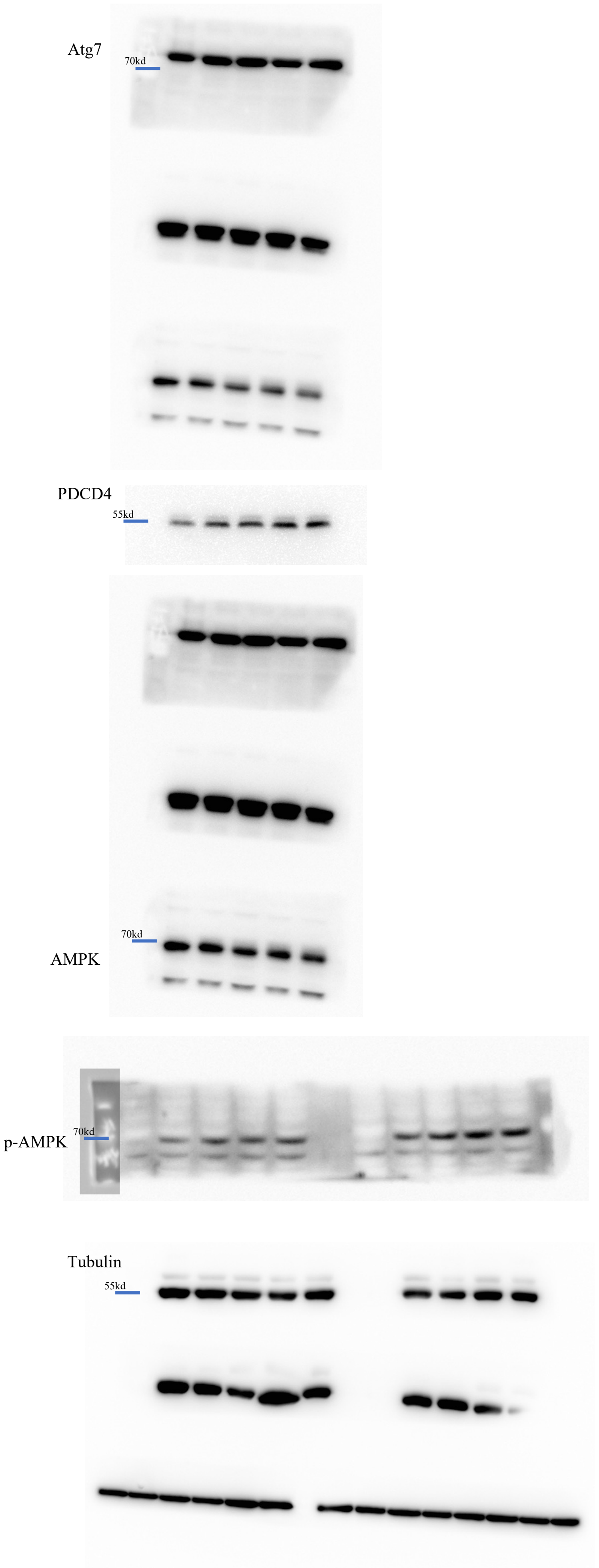

Supplementary Figure 1c

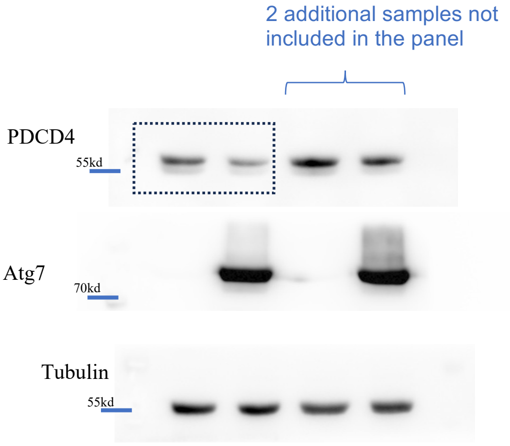

Supplementary Figure 2e

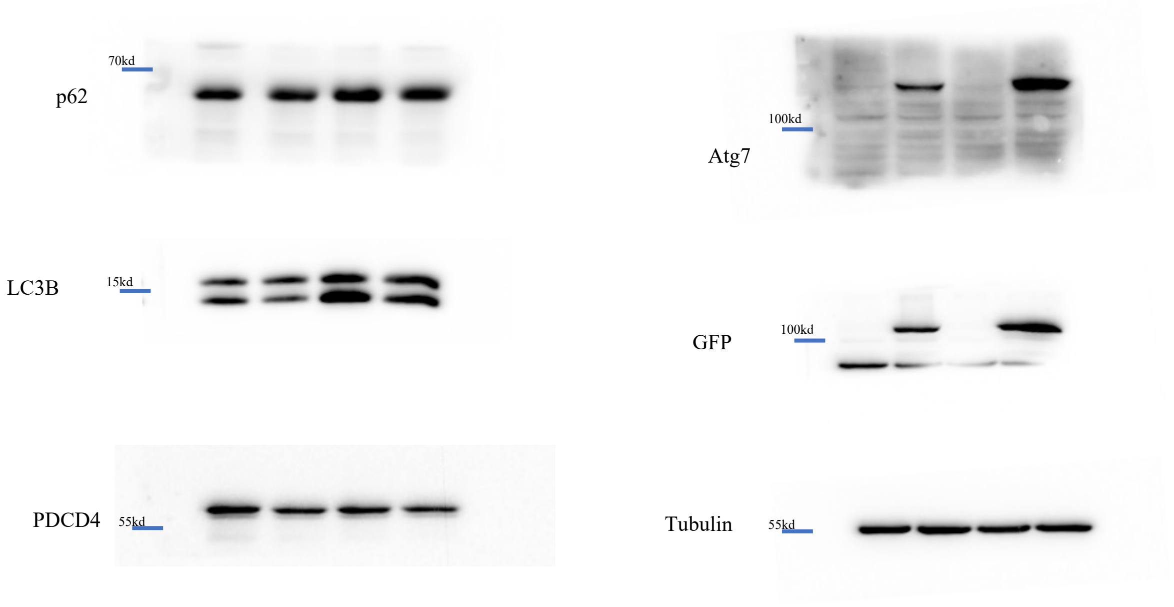

Supplementary Figure 3a

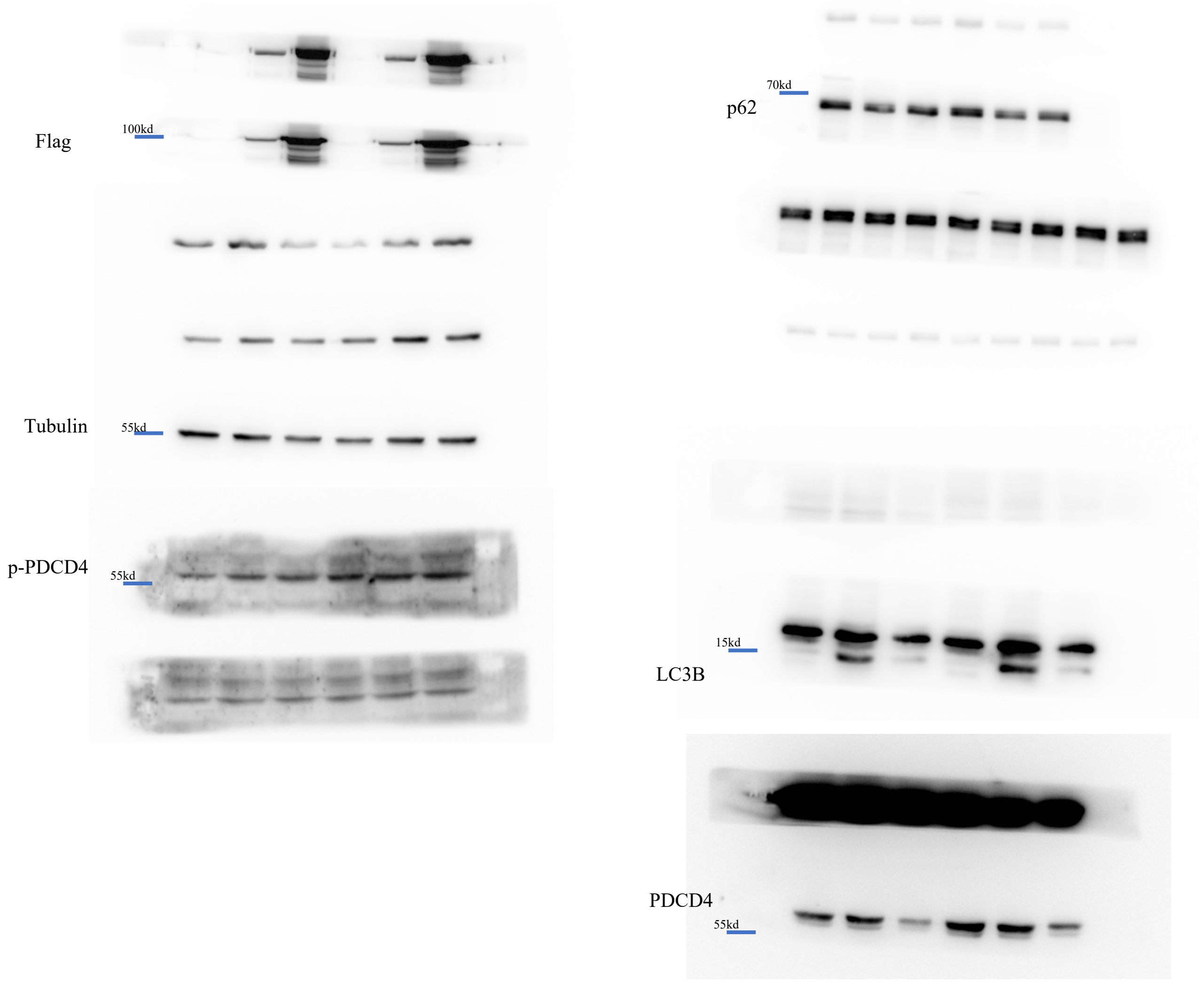

Supplementary Figure 3b

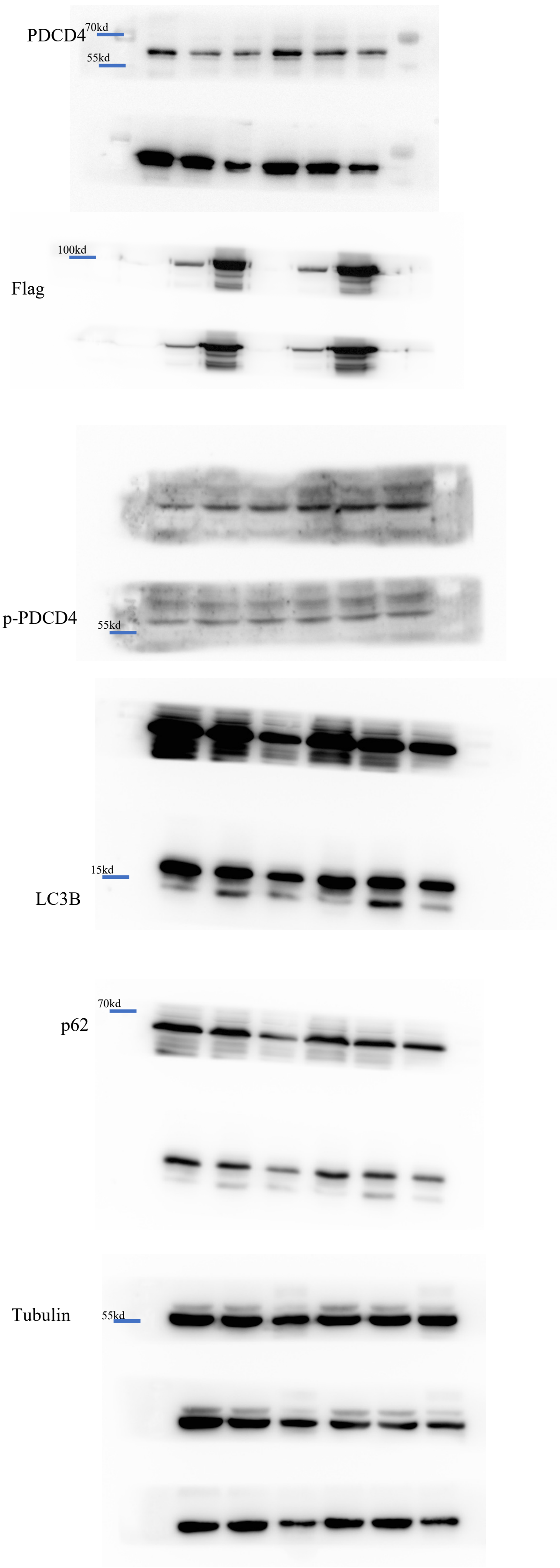

Supplementary Figure 3c

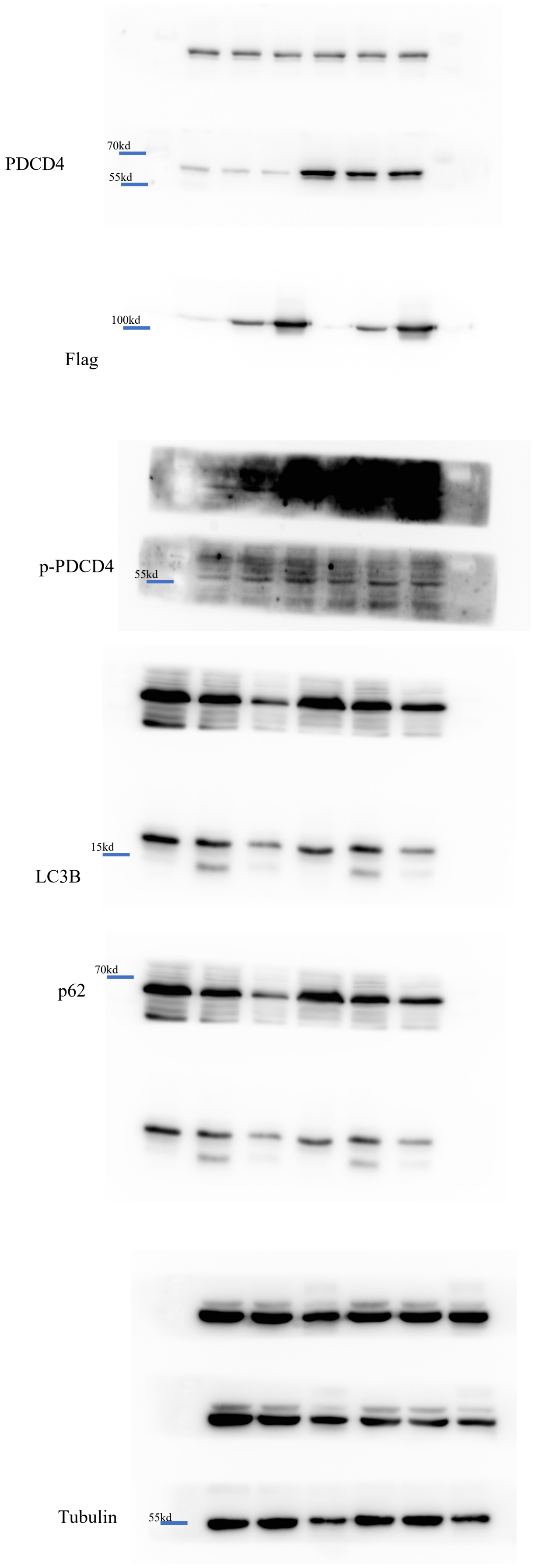

Supplement: Supplementary file 1 — Supplementary Information [file 42003_2023_5656_MOESM1_ESM.pdf]
