# Supplementary material for: Atg7 senses ATP levels and regulates AKT1-PDCD4 phosphorylation-ubiquitination axis to promote survival during metabolic stress
Source: Commun Biol. 2023 Dec 11;6:1252. doi: 10.1038/s42003-023-05656-7 (PMC10713595; doi:10.1038/s42003-023-05656-7)
Supplement: Supplementary file 2 — Description of Additional Supplementary Files [file 42003_2023_5656_MOESM2_ESM.pdf]

## Description of Additional Supplementary Files

**File name:** Supplementary Data 1

**Description:** The numerical source data underlying all graphs.
