# Supplementary material for: Atg7 senses ATP levels and regulates AKT1-PDCD4 phosphorylation-ubiquitination axis to promote survival during metabolic stress
Source: Commun Biol. 2023 Dec 11;6:1252. doi: 10.1038/s42003-023-05656-7 (PMC10713595; doi:10.1038/s42003-023-05656-7)
Supplement: Supplementary file 4 — Reporting Summary [file 42003_2023_5656_MOESM4_ESM.pdf]

nature portfolio

Corresponding author(s): Liu Cao

Last updated by author(s): Nov 21, 2023

Reporting Summary

Nature Portfolio wishes to improve the reproducibility of the work that we publish. This form provides structure for consistency and transparency in reporting. For further information on Nature Portfolio policies, see our Editorial Policies and the Editorial Policy Checklist.

Statistics

For all statistical analyses, confirm that the following items are present in the figure legend, table legend, main text, or Methods section.

n/a	Confirmed	
☐	☒	The exact sample size (*n*) for each experimental group/condition, given as a discrete number and unit of measurement
☐	☒	A statement on whether measurements were taken from distinct samples or whether the same sample was measured repeatedly
☐	☒	The statistical test(s) used AND whether they are one- or two-sided<br>*Only common tests should be described solely by name; describe more complex techniques in the Methods section.*
☒	☐	A description of all covariates tested
☐	☒	A description of any assumptions or corrections, such as tests of normality and adjustment for multiple comparisons
☐	☒	A full description of the statistical parameters including central tendency (e.g. means) or other basic estimates (e.g. regression coefficient) AND variation (e.g. standard deviation) or associated estimates of uncertainty (e.g. confidence intervals)
☐	☒	For null hypothesis testing, the test statistic (e.g. *F*, *t*, *r*) with confidence intervals, effect sizes, degrees of freedom and *P* value noted<br>*Give P values as exact values whenever suitable.*
☒	☐	For Bayesian analysis, information on the choice of priors and Markov chain Monte Carlo settings
☒	☐	For hierarchical and complex designs, identification of the appropriate level for tests and full reporting of outcomes
☒	☐	Estimates of effect sizes (e.g. Cohen's *d*, Pearson's *r*), indicating how they were calculated

Our web collection on statistics for biologists contains articles on many of the points above.

Software and code

Policy information about availability of computer code

Data collection	Flow cytometry date collection with BD Accuri C6<br>Confocal imaging collection with Nikon, Ti-E, DS-Ri2<br>Cell fluorescence image collection with Nikon eclipse 80i<br>Western blot image collection with DNR MicroChemi 4,2
Data analysis	The flow cytometry date were analyzed using FlowJo Version 10.4 software.<br>Western blot date were analyzed using ImageJ java software.<br>The statistical analysis was performed using GraphPad Prism Version 9.4.1 software.<br>Figure preparation was performed using Illustrator 2022.

For manuscripts utilizing custom algorithms or software that are central to the research but not yet described in published literature, software must be made available to editors and reviewers. We strongly encourage code deposition in a community repository (e.g. GitHub). See the Nature Portfolio guidelines for submitting code & software for further information.

Data

Policy information about availability of data

All manuscripts must include a data availability statement. This statement should provide the following information, where applicable:

- Accession codes, unique identifiers, or web links for publicly available datasets
- A description of any restrictions on data availability
- For clinical datasets or third party data, please ensure that the statement adheres to our policy

All additional datasets referred to in the current study are available from the corresponding authors on request. Source data are provided in the Source Data file.

Research involving human participants, their data, or biological material

Policy information about studies with human participants or human data. See also policy information about sex, gender (identity/presentation), and sexual orientation and race, ethnicity and racism.

Reporting on sex and gender	N/A
Reporting on race, ethnicity, or other socially relevant groupings	N/A
Population characteristics	N/A
Recruitment	N/A
Ethics oversight	N/A

Note that full information on the approval of the study protocol must also be provided in the manuscript.

Field-specific reporting

Please select the one below that is the best fit for your research. If you are not sure, read the appropriate sections before making your selection.

☒ Life sciences ☐ Behavioural & social sciences ☐ Ecological, evolutionary & environmental sciences

For a reference copy of the document with all sections, see nature.com/documents/nr-reporting-summary-flat.pdf

Life sciences study design

All studies must disclose on these points even when the disclosure is negative.

Sample size	No sample size calculation was performed
Data exclusions	No data were excluded from the analyses
Replication	All results were expressed as mean ± standard deviation (Mean ± SD) from at least three independent experiments. Trend is reproducible.
Randomization	Cells were not randomly allocated into experiment groups as this was not required. To help control covariates, experimental were typically performed on the same day. In addition, cells imaged during confocal were randomly sampled as we could not control which cells landed in the field of view. Images of cells were chosen at random and all cells within the field of view (except those on the edge) were used for analysis.
Blinding	Image acquisition from different cell lines or with different conditions were not blinded but relied on unbiased data collection from random regions in the wells or coverslip. Furthermore, all cells in a field of view (excep those on the edge of a field of view) were analyzed.

Reporting for specific materials, systems and methods

We require information from authors about some types of materials, experimental systems and methods used in many studies. Here, indicate whether each material, system or method listed is relevant to your study. If you are not sure if a list item applies to your research, read the appropriate section before selecting a response.

Materials & experimental systems

n/a	Involved in the study
☐	☒ Antibodies
☐	☒ Eukaryotic cell lines
☒	☐ Palaeontology and archaeology
☒	☐ Animals and other organisms
☒	☐ Clinical data
☒	☐ Dual use research of concern
☒	☐ Plants

Methods

n/a	Involved in the study
☒	☐ ChIP-seq
☐	☒ Flow cytometry
☒	☐ MRI-based neuroimaging

Antibodies

Antibodies used

PDCD4 antibody (IP:1:200, IB:1:1000, Santa Cruz Biotechnology, sc-376430; Cell Signaling Technology, #9535); phospho-PDCD4(Ser67) antibody (IB: 1:1000, Abcam, ab73343; Sangon Biotech, D151427); Atg7 antibody (IP:1:200, IB:1:1000, Sigma, SAB1407006); AKT antibody (IP:1:200, IB:1:1000, Cell Signaling Technology, #4691); AMPKα (IB:1:1000, Cell Signaling Technology, #2532); Phospho-AMPKα(Thr172)(IB:1:1000, Cell Signaling Technology, #2531), Caspase3 antibody (IB:1:1000,Cell Signaling Technology, #9662); Cleaved PARP antibody (IB:1:1000, Cell Signaling Technology, #5625); Ubiquitin antibody (IB:1:1000, Cell Signaling Technology, #3933); HA-Tag antibody (IB:1:1000, Cell Signaling Technology, #3724); Flag antibody (IB:1:1000, Genomics Technology, SG4110-16); GFP antibody (IB:1:1000, GenScript, A01388-40); α-Tubulin antibody (IB:1:2000, Cell Signaling Technology, #2144);

Validation

Validation provided by supplier.

Eukaryotic cell lines

Policy information about cell lines and Sex and Gender in Research

Cell line source(s)

HCT 116 (ATCC), HEK 293 (ATCC).

Authentication

The parental HCT 116 and HEK 293 cells were authenticated by the supplier (ATCC).

Mycoplasma contamination

Cell lines were routinely tested and found negative for mycoplasma contamination.

Commonly misidentified lines
(See ICLAC register)

No commonly misidentified cell lines were used.

Flow Cytometry

Plots

Confirm that:

☒ The axis labels state the marker and fluorochrome used (e.g. CD4-FITC).

☒ The axis scales are clearly visible. Include numbers along axes only for bottom left plot of group (a 'group' is an analysis of identical markers).

☐ All plots are contour plots with outliers or pseudocolor plots.

☐ A numerical value for number of cells or percentage (with statistics) is provided.

Methodology

Sample preparation

For cell apoptosis assay, cells were transfected with siRNA. After24hours, the experimental groups were subjected to ATP depletion for 48hours. Then, the frequency of apoptotic cells was tested using an Annexin V-FITC/PI kit (KeyGENBioTECH, KGA108) following the manufacturer's standard procedures.

Instrument

BD Accuri C6

Software

The date were analyzed using FlowJo Version 10.4 software, and the statistical analysis was performed using GraphPad Prism Version 9.4.1 software.

Cell population abundance

Any sorted cells were only used if they were >99% abundant. This was determined by staining with fluorescently-tagged antibodies and analyzing cells by flow cytometry.

Gating strategy

HCT116 cell lines and HEK293 cells were first identified based on FSC-A/SSC-A. In the case of apoptosis , cells were first gating strategy for isolating single cells. The gated cells were then used to produce the flow histograms seen in the figures. Positive cells are defined by any signal above an isotype control/unstained cells.

☒ Tick this box to confirm that a figure exemplifying the gating strategy is provided in the Supplementary Information.
